# Cryptic Splicing of *GAP43* mRNA is a Novel Hallmark of TDP‐43‐Associated ALS and AD

**DOI:** 10.1002/advs.202412054

**Published:** 2025-06-29

**Authors:** Mingming Yang, Qi Wang, Dongkun Kang, Shijia Wang, Yanli Jiang, Jian‐Zhi Wang, Chen Ming, Rong Liu, Jianlan Gu, Xiaochuan Wang

**Affiliations:** ^1^ Department of Pathophysiology School of Basic Medicine Key Laboratory of Education Ministry/Hubei Province of China for Neurological Disorders Tongji Medical College Huazhong University of Science and Technology Wuhan Hubei 430030 China; ^2^ Department of Biochemistry and Molecular Biology School of Medicine Key Laboratory of Neuroregeneration and Ministry of Education of Jiangsu Co‐innovation Center of Neuroregeneration Nantong University Nantong Jiangsu 226001 China; ^3^ Department of Public Health and Medicinal Administration Faculty of Health Sciences University of Macau Macao SAR 999078 China; ^4^ Hubei Key Laboratory of Cognitive and Affective Disorders Institute of Biomedical Sciences School of Medicine Jianghan University Wuhan 430056 China

**Keywords:** cryptic exon, GAP43, mis‐splicing, TDP‐43

## Abstract

Cytoplasmic aggregation of transactive response DNA‐binding protein 43 (TDP‐43) is a hallmark of amyotrophic lateral sclerosis (ALS) and occurs in 57% of Alzheimer's disease (AD) cases. TDP‐43 regulates RNA processing, including cryptic exon splicing. Here, we demonstrate that TDP‐43 directly controls growth‐associated protein (GAP43) expression by binding to its pre‐mRNA. Loss or hyperphosphorylation of TDP‐43 disrupts this binding, leading to the inclusion of cryptic exon *4a1*, which introduces premature stop codons and reduces GAP43 protein levels. RNA sequencing analysis of ALS and AD brains revealed *GAP43* downregulation, while *4a1* is upregulated in AD cases with phosphorylated TDP‐43. TDP‐43 knockdown impaired axonal regeneration in induced pluripotent stem cell (iPSC)‐derived motor neurons, whereas GAP43 restoration rescued this defect. These findings suggest that the loss of GAP43 contributes to neurodegeneration in ALS and AD. The inclusion of *GAP43* cryptic exon *4a1* may serve as a hallmark of TDP‐43 proteinopathies, highlighting a mechanistic link between TDP‐43 dysfunction and neuronal vulnerability.

## Graphical abstract

TDP‐43 dysfunction disrupts RNA processing, inducing cryptic exon *4a1* inclusion in *GAP43* and reducing its protein levels. This aberrant splicing impairs axonal regeneration and contributes to neurodegeneration in ALS and AD. RNA‐seq of patient brains reveals *GAP43* downregulation and *4a1* upregulation, identifying cryptic exon *4a1* as a potential molecular hallmark of TDP‐43 proteinopathies.

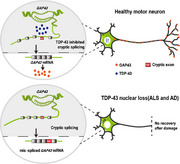

## 1 Introduction

Transactive response DNA‐binding protein 43 (TDP‐43) is a widely expressed RNA‐binding protein encoded by the *TARDBP* gene. Under normal conditions, TDP‐43 localizes to nuclei and plays critical roles in RNA processing, including transcription, pre‐mRNA splicing, and RNA transport.^[^ ^1^, ^2^, ^3^, ^4^ ^]^ One of its key functions is binding to intronic regions to suppress the inclusion of cryptic exons.^[^ ^2^, ^5^ ^]^ Cryptic exons, which are normally retained within introns, do not appear in mature mRNA under physiological conditions. However, TDP‐43 depletion leads to the aberrant splicing of cryptic exons into mRNA, causing frameshifts, premature translational termination, and reduced RNA stability.^[^ ^6^, ^7^ ^]^

Among known TDP‐43‐regulated cryptic splicing events, Stathmin like 2 (STMN2) and UN‐coordinated 13 homolog A protein (UNC13A) have received considerable attention. STMN2, a neuronal protein that regulates microtubule stability, contains a cryptic exon *2a* (*CE2a*) that is normally excluded from mature *STMN2* mRNA.^[^ ^8^, ^9^, ^10^ ^]^ However, TDP‐43 depletion or functional impairment results in *CE2a* inclusion, introducing a premature stop codon and a polyadenylation signal, leading to a significant reduction in *STMN2* mRNA levels.^[^ ^9^ ^]^ Similarly, *UNC13A*, a high‐risk gene for amyotrophic lateral sclerosis (ALS) and frontotemporal dementia (FTD), harbors a cryptic exon between exons 20 and 21. When TDP‐43 is lost or dysfunctional, this cryptic exon is aberrantly spliced into mature *UNC13A* mRNA, introducing multiple stop codons.^[^ ^7^ ^]^ However, UNC13A downregulation only occurs when TDP‐43 knockdown exceeds 80% efficiency.^[^ ^11^ ^]^ Notably, mis‐splicing of *STMN2* and *UNC13A* also occurs in Alzheimer's disease (AD) brains with pathological phosphorylated TDP‐43 (pTDP‐43).^[^ ^12^ ^]^ Given their involvement in neurodegeneration, *STMN2* and *UNC13A* are being explored as potential therapeutic targets and biomarkers for TDP‐43 proteinopathies, including ALS and FTD.^[^ ^6^, ^9^, ^13^ ^]^ However, the role of other TDP‐43‐regulated cryptic splicing events in neurodegenerative disease pathogenesis remains unclear.

Growth‐associated protein 43 (GAP43) is a fast‐translocating cytosolic phosphoprotein critical for neuronal development, axonal regeneration, and synaptic remodeling, serving as an intrinsic determinant of neuronal plasticity.^[^ ^14^, ^15^, ^16^ ^]^ Its specific expression in the nervous system, particularly in neurons, has been well documented. GAP43 is widely distributed in neurons of the cerebrum, cerebellum, spinal cord, dorsal root ganglia, and autonomic nervous system and is predominantly expressed along the entire axon of developing neurons, especially in growth cones and specific nerve terminal regions of mature neurons.^[^ ^17^, ^18^, ^19^, ^20^ ^]^ During early mammalian central nervous system (CNS) development, GAP43 is highly expressed in axons and synapses but declines significantly as neurons mature and synaptic connections are established.^[^ ^21^ ^]^

Neuronal degeneration and loss are pathological hallmarks of ALS and AD,^[^ ^22^, ^23^ ^]^ whereas GAP43 promotes neuronal growth and repair.^[^ ^24^ ^]^ In this study, we found that *GAP43* mRNA was significantly downregulated following TDP‐43 depletion in cortical neurons capable of differentiation, as well as in AD brains with pathological pTDP‐43. Notably, *GAP43* mis‐splicing occurred in a TDP‐43 dose‐dependent manner. Mechanistically, the RNA recognition motif 1 (RRM1) of TDP‐43 binds to cryptic exon *4a1* of *GAP43*, preventing its mis‐splicing. However, aberrant TDP‐43 phosphorylation enhances cryptic exon *4a1* inclusion, leading to GAP43 downregulation. Functionally, inhibition of either TDP‐43 or GAP43 in retinoic acid (RA)‐treated M17 cells suppressed axonal growth cone development. Importantly, restoring GAP43 expression after TDP‐43 depletion rescued axonal growth cone formation in M17 cells, and reversed axonal regeneration defects in induced pluripotent stem cell (iPSC)‐derived motor neurons, suggesting that GAP43 restoration could be a potential therapeutic strategy for TDP‐43 proteinopathies, particularly ALS and AD.

## 2 Results

### 2.1 TDP‐43 Knockdown Affects the Expression of Axonogenesis‐related Genes

**Figure 1:**
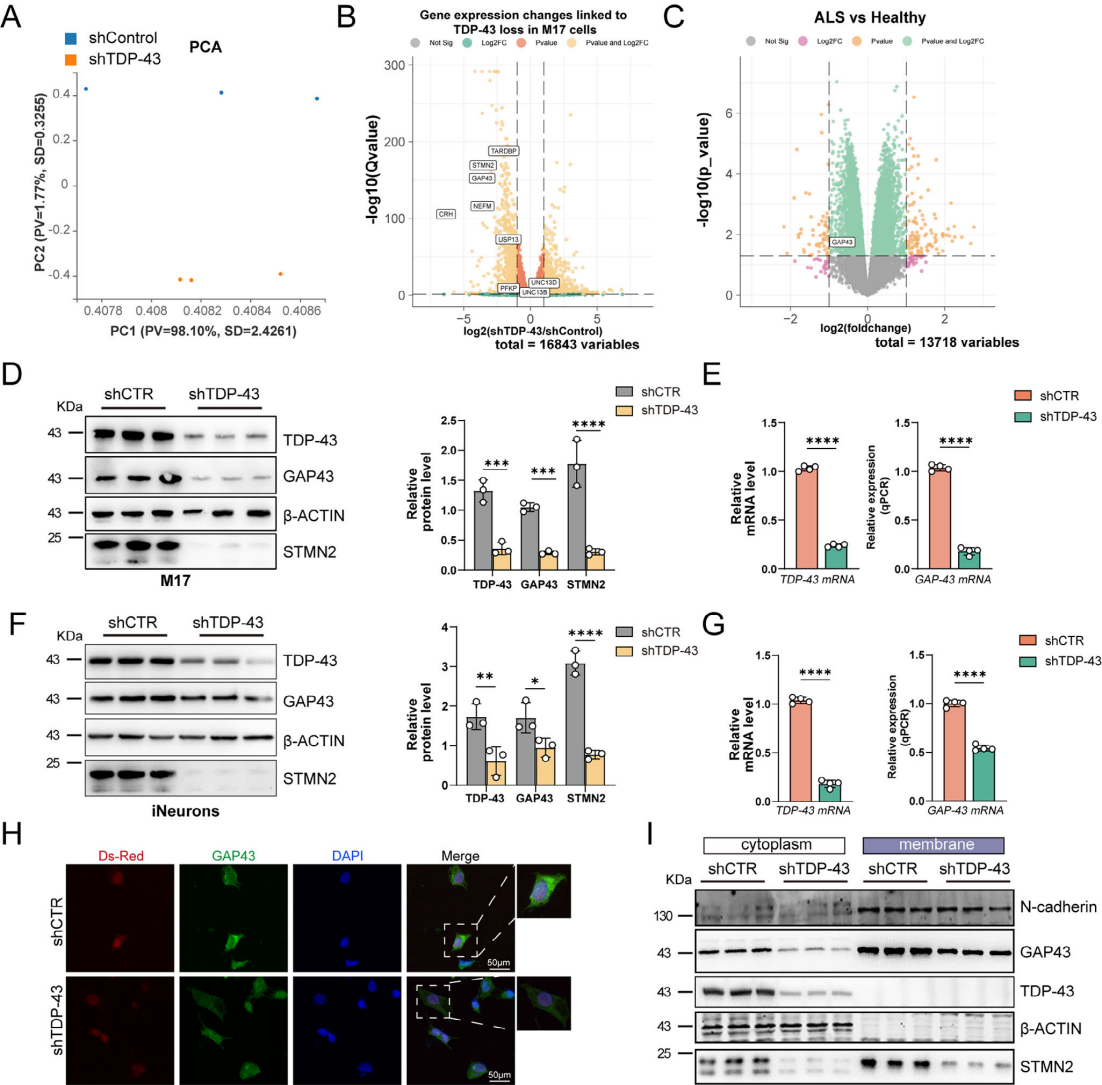
The expression of GAP43 is decreased in TDP‐43 knockdown cells and ALS patients. A) PCA plot showing the clear separation of shTDP43 in M17 cells. B) Volcano plot showing the gene expression in shTDP‐43 M17 cells. C) Volcano plot showing the expression of GAP43 in ALS patients based on 13718 variable genes generated from dataset GSE122261. D) Western blotting analysis of the lysate of shTDP‐43 M17 cells (n = 3), and quantification of TDP‐43, GAP43 and STMN2 proteins. Data are mean ± s.e.m.; ^***^ *P* < 0.001; ^****^ *P* < 0.0001 for shTDP‐43 versus shCTR (Two‐Way ANOVA, Sidak's multiple comparison). E) qPCR analysis of *TDP‐43* and *GAP43* transcripts in shTDP‐43 M17 cells (n = 4). Data are mean ± s.e.m.; ^****^ *P* < 0.0001 for shTDP‐43 versus shCTR (Unpaired two‐tailed Student's *t* test). F) Western blotting analysis of the lysate of shTDP‐43 iNeurons (n = 3), and quantification of TDP‐43, GAP43 and STMN2 proteins. iNeurons, iPSC‐derived cortical neurons. Data are mean ± s.e.m.; ^*^ *P* < 0.05; ^**^ *P* < 0.01; ^****^ *P* < 0.0001 for shTDP‐43 versus shCTR (Two‐Way ANOVA, Sidak's multiple comparison). G) qPCR analysis of *TDP‐43* and *GAP43* transcripts in shTDP‐43 iNeurons (n = 4). Data are mean ± s.e.m.; ^****^ *P* < 0.0001 for shTDP‐43 versus shCTR (Unpaired two‐tailed Student's *t* test). H) Immunofluorescence staining of GAP43 in shTDP‐43 M17 cells (Scale bar, 50 µm). I) Western blotting analysis of the cytoplasm and cytomembrane fractionation in shTDP‐43 M17 cells.

Neuronal loss is a key pathological feature of ALS and AD.^[^ ^22^, ^23^ ^]^ Accumulating evidence strongly supports that TDP‐43 dysfunction contributes to neuronal injury,^[^ ^25^, ^26^, ^27^, ^28^ ^]^ yet the precise molecular mechanisms remain unclear. To investigate these mechanisms, we treated M17 cells with lentivirus expressing shTDP‐43, followed by transcriptome sequencing (RNA‐seq) and bioinformatic analysis. Principal component analysis (PCA) revealed substantial heterogeneity between the control (shCTR) and TDP‐43 knockdown (shTDP‐43) groups (**Figure** **1**A). Kyoto Encyclopedia of Genes and Genomes (KEGG) analysis highlighted enrichment in pathways related to ALS, AD, and axon guidance (Figure S1A, Supporting Information). Gene Ontology (GO) analysis further indicated that genes associated with synapse development, motor neuron axonal guidance, axon genesis, and microtubule‐associated complexes were significantly downregulated in the TDP‐43 knockdown group (Figure S1B, Supporting Information). Functional enrichment analysis revealed that differentially expressed genes were primarily involved in mRNA binding and calmodulin interactions (Figure S1B, Supporting Information). Collectively, these findings suggest that TDP‐43 dysfunction is closely associated with neuronal loss and axonogenesis.

To further explore the link between TDP‐43 and axonogenesis, we performed hierarchical clustering of the top 40 axonogenesis‐related genes and found that *GAP43* was the most significantly downregulated gene in the TDP‐43 knockdown group (Figure S1C, Supporting Information). Additionally, Gene Set Enrichment Analysis (GSEA) revealed that the neuronal regeneration pathway was significantly downregulated, with GAP43 exhibiting the most pronounced reduction. Furthermore, the GO chord diagram indicated that GAP43 plays crucial roles in axon generation, neuronal guidance, calcium binding, and neuronal recognition pathways (Figure S1D, E, Supporting Information). These findings suggest that GAP43 may be a key mediator of synaptic dysfunction following TDP‐43 depletion. Quantitative PCR (qPCR) validation of the nine most significantly altered genes confirmed the RNA‐seq results (Figure S1F, Supporting Information).

Volcano plot analysis of 16843 genes from the RNA‐seq dataset identified significant downregulation of *STMN2*, phosphofructokinase platelet (*PFKP*), ubiquitin‐specific peptidase 13 (*USP13*), and neurofilament middle chain (*NEFM*) in the TDP‐43 knockdown group (Figure 1B), consistent with previous reports.^[^ ^29^, ^30^ ^]^ In contrast, no change in *UNC13A* expression was detected^[^ ^6^, ^31^ ^]^ (Figure 1B), likely due to the moderate knockdown efficiency of TDP‐43. Notably, previous studies have shown that *UNC13A* downregulation occurs only when TDP‐43 knockdown efficiency exceeds 80%.^[^ ^11^ ^]^ We validated the knockdown efficiency of TDP‐43 in M17 cells by qPCR and determined it to be ≈60% (Figure S2A, Supporting Information), which likely accounts for the absence of a detectable reduction in *UNC13A* mRNA expression in our dataset. In contrast, in iPSC‐derived cortical neurons (iNeurons) with a knockdown efficiency of 80%, we observed a reduction in the full‐length transcripts of both *UNC13A* and *STMN2* and the occurrence of cryptic splicing events (Figure S2B, Supporting Information). Importantly, volcano plot analysis identified *GAP43* as the most significantly downregulated gene. Consistently, analysis of publicly available RNA‐seq data from ALS patients also revealed a significant reduction in GAP43 expression (Figure 1B,C), suggesting a strong association between GAP43, TDP‐43 dysfunction, and ALS pathology.

To further validate these findings, we knocked down TDP‐43 in M17 cells and iNeurons and assessed the expression of STMN2 and GAP43. Both genes exhibited significant reductions at both the mRNA and protein levels (Figure 1D–G). Immunofluorescence staining in M17 cells confirmed that GAP43 expression was markedly reduced in the shTDP‐43 group (Figure 1H). Given that GAP43 is localized in both the cytoplasm and cytomembrane,^[^ ^14^ ^]^ we performed subcellular fractionation in shTDP‐43 M17 cells and found that GAP43 was significantly reduced in both compartments following TDP‐43 knockdown (Figure 1I). These results collectively indicate that TDP‐43 regulates GAP43 expression and its subcellular localization, particularly its transport to the cytomembrane.

### 2.2 TDP‐43 Represses the Mis‐splicing of *GAP43* Cryptic Exon *4a1*

**Figure 2:**
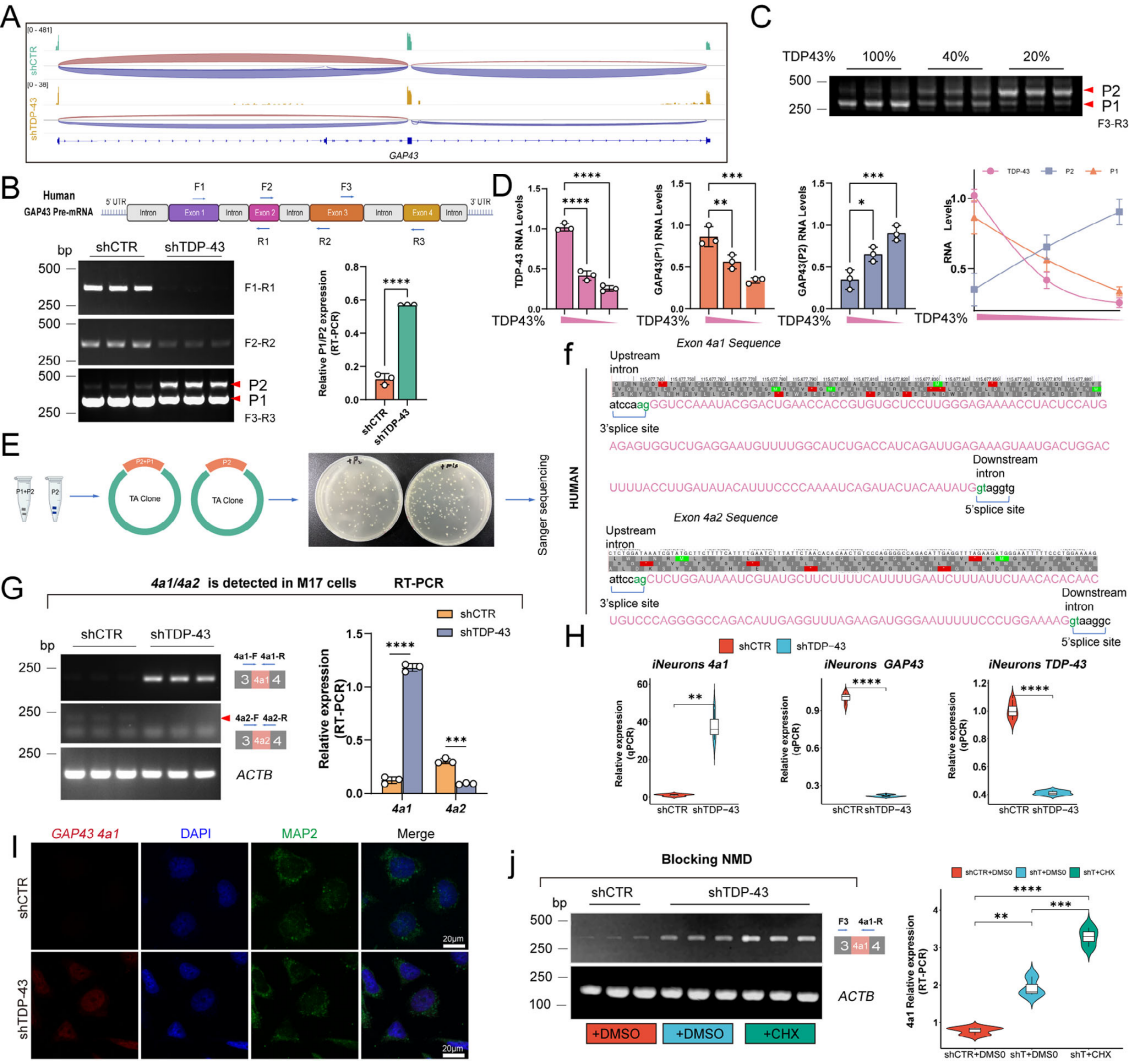
*GAP43* produces cryptic exon *4a1* in TDP‐43 knockdown M17 cells and iNeurons. A) Visualization of RNA‐seq reads mapping to *GAP43* from shTDP‐43 M17 cells. B) Schematic diagram of human *GAP43* pre‐mRNA with 4 exons (purple, red, orange, and yellow, respectively) and introns (grey). Arrows are forward (F) and reverse (R) primers. The splicing products of *GAP43* in shTDP‐43 M17 cells were analyzed by RT‐PCR with F1‐R1, F2 ‐ R2 and F3‐R3 primers and quantitated (n = 3). Data are mean ± S.D.; ^****^ *P* < 0.0001 for shTDP‐43 versus shCTR (Unpaired two‐tailed Student's *t* test). C,D), The splicing products of *GAP43* in shTDP‐43 M17 cells were analyzed by RT‐PCR with F3‐R3 primers (C), quantification, and the correlation of TDP‐43, GAP43 (P1), and GAP43 (P2) with TDP‐43 knockdown efficiency (D) (n = 3). Data are mean ± S.D.; ^***^ *P* < 0.001; ^****^ *P* < 0.0001 for shTDP‐43 versus shCTR (One‐Way ANOVA, Tukey's multiple comparison). E,F), P2 identified by TA cloning (E) and the sequence results of sanger sequencing (F) compared to the intron between exon 3 and exon 4 in *GAP43* gene. G), The splicing products of *GAP43 4a1* and *4a2* in shTDP‐43 M17 cells were analyzed by RT‐PCR and quantitated (n = 3). Data are mean ± S.D.; ^***^ *P* < 0.001; ^****^ *P* < 0.0001 for shTDP‐43 versus shCTR (Two‐Way ANOVA, Sidak's multiple comparison). H), The splicing products of *GAP43 4a1* in shTDP‐43 iNeurons were analyzed by RT‐PCR and quantitated in a violin plot (n = 3). Data are mean ± S.D.; ^**^ *P* < 0.01; ^****^ *P* < 0.0001 for shTDP‐43 versus shCTR (Unpaired two‐tailed Student's *t* test). I), Representative images of RNA‐FISH with *GAP43 4a1* probe in shTDP‐43 M17 cells. Scale bar, 20 µm. J), The splicing products of *GAP43 4a1* in shTDP‐43 M17 cells with CHX treatment (blocking NMD) were analyzed by RT‐PCR, and quantitated in a violin plot (n = 3). Data are mean ± S.D.; ^**^ *P* < 0.01; ^***^ *P* < 0.001; ^****^ *P* < 0.0001 for shTDP‐43 versus shCTR (One‐Way ANOVA, Tukey's multiple comparison).

One of the major functions of TDP‐43 is the suppression of cryptic exon inclusion during RNA splicing.^[^ ^2^ ^]^ To investigate whether *GAP43* undergoes cryptic splicing upon TDP‐43 knockdown, we performed splicing analysis using Integrative Genomics Viewer (IGV) on our RNA‐seq dataset. Consistent with previous reports, we detected the inclusion of cryptic exon *2a* in *STMN2* (Figure S2C, Supporting Information). Notably, we also observed an aberrant sequencing peak between exons 3 and 4 of *GAP43* (**Figure** **2**A), suggesting a novel splicing event induced by TDP‐43 depletion.

To validate this mis‐splicing event, we designed three primer pairs (F1‐R1, F2‐R2, and F3‐R3) spanning the four exons of *GAP43* and performed RT‐PCR in TDP‐43 knockdown M17 cells. Interestingly, the F3‐R3 primer pair amplified an additional P2 band of ~500 bp (Figure 2B). Sequence alignment of P2 against the genome revealed that it was absent in normal *GAP43* transcripts, indicating the presence of cryptic splicing. Previous studies have shown that the mis‐splicing of *STMN2* and *UNC13A* exhibits varying susceptibility to TDP‐43 depletion.^[^ ^6^, ^7^, ^9^ ^]^ To determine whether *GAP43* mis‐splicing follows a similar pattern, we generated a stable M17 cell line with a doxycycline (DOX)‐inducible TDP‐43 knockdown system and verified knockdown efficiency at different DOX concentrations (Figure S2D, Supporting Information). Notably, *GAP43* mis‐splicing was detectable when TDP‐43 knockdown reached 40% and became more pronounced at 80% knockdown (Figure 2C,D), suggesting that *GAP43* mis‐splicing is TDP‐43‐dependent. To identify the sequence of the P2 band, we performed gel DNA purification, TA cloning, and Sanger sequencing. Alignment analysis revealed two cryptic exon variants, *4a1* (160 bp) and *4a2* (120 bp), both originating from the intronic region between exons 3 and 4 of *GAP43*. Importantly, both sequences adhered to the canonical AG‐GU splice donor‐acceptor rule, confirming their role as cryptic exons (Figure 2E,F).^[^ ^32^ ^]^ Further analysis revealed multiple stop codons within *GAP43 4a1* and *4a2* (Figure S2F, Supporting Information). By designing specific primers for these cryptic exons and performing RT‐PCR, we found that *GAP43 4a1* was significantly upregulated, whereas *4a2* was downregulated in TDP‐43 knockdown M17 cells (Figure 2G). Given its pronounced increase, we focused on *GAP43 4a1* in subsequent experiments.

Since M17 cells are neuroblastoma‐derived, we next sought to validate these findings in human neurons. We differentiated iPSCs into cortical neurons over four weeks (Figure S2E, Supporting Information) and assessed *GAP43 4a1* expression by RT‐qPCR. Consistent with our M17 cell data, *4a1* levels were significantly upregulated in TDP‐43 knockdown iNeurons (Figure 2H). Interestingly, homology alignment in the UCSC genome browser revealed that *4a1* and *4a2* sequences are highly conserved in primates but not in rodents (Figures S3 and S4, Supporting Information). To further explore species‐specific differences, we knocked down TDP‐43 in murine N2a cells and found that while GAP43 and STMN2 protein levels remained unchanged, *GAP43* mRNA levels were paradoxically increased (Figure S2G, Supporting Information), suggesting a distinct splicing regulation mechanism in rodents.

To visualize *GAP43 4a1* inclusion at the single‐cell level, we performed fluorescence in situ hybridization (FISH) using a specific probe targeting *4a1* in M17 cells. As expected, *4a1* signals were detected only in the TDP‐43 knockdown group (Figure 2I), further supporting its role as a cryptic exon induced by TDP‐43 loss. Given that *4a1* contains multiple premature stop codons, its inclusion is likely to trigger nonsense‐mediated mRNA decay (NMD), leading to *GAP43* mRNA degradation and reduced expression. Indeed, when we inhibited NMD using CHX,^[^ ^33^ ^]^ *GAP43 4a1* levels were further elevated in the TDP‐43 knockdown group (Figure 2J). Collectively, our results indicate that TDP‐43 depletion promotes the cryptic splicing of *GAP43 4a1*, leading to mRNA degradation and subsequent loss of GAP43 expression.

### 2.3 TDP‐43 RRM1 Directly Binds to *GAP43 4a1* and Represses Cryptic Exon Inclusion

**Figure 3:**
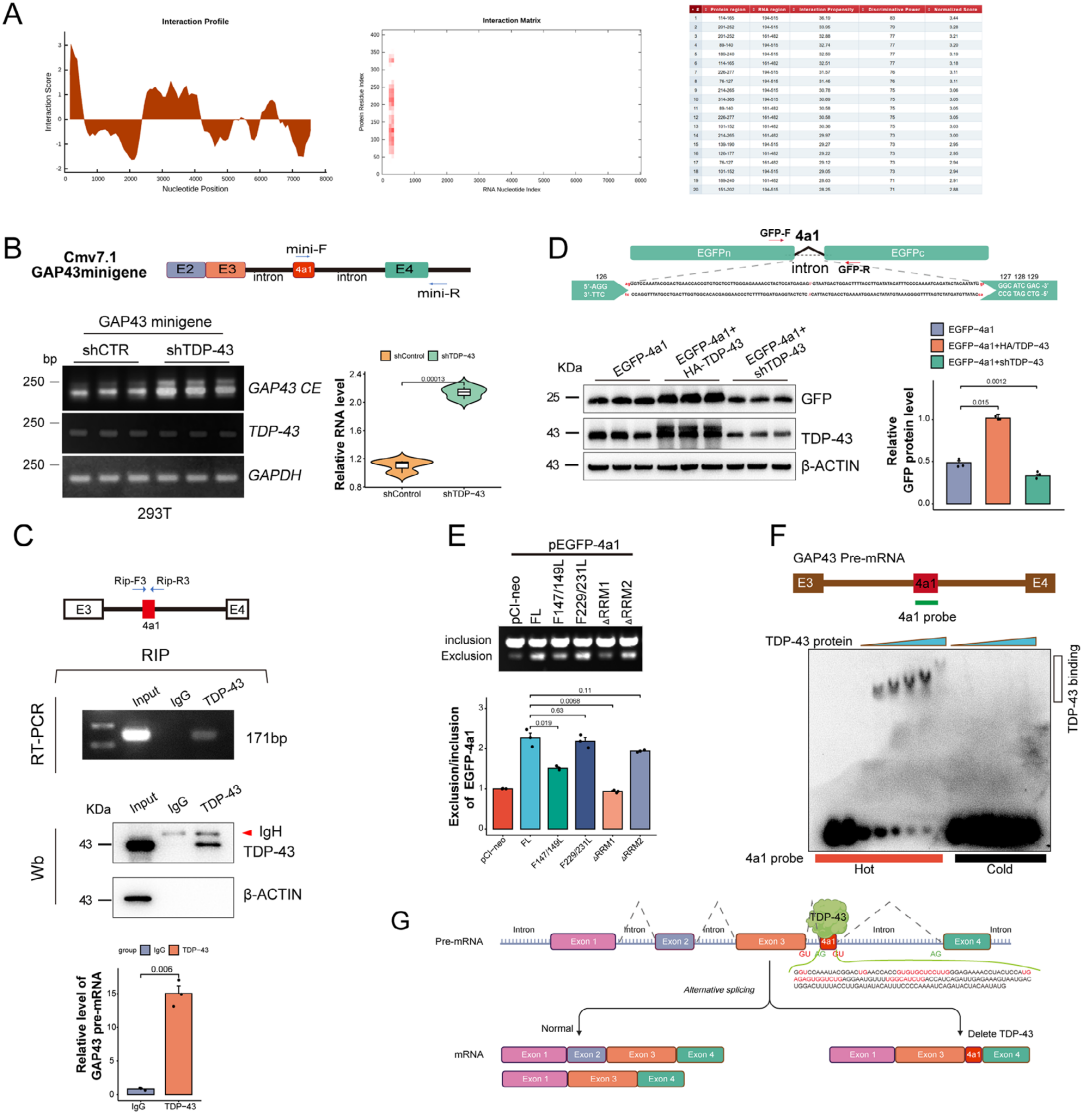
TDP‐43 inhibits the production of *GAP43* cryptic exon through RRM1. A) Predicted binding sites for each peptide of TDP‐43 protein and *GAP43* pre‐mRNA (containing chr3: 115, 676, 246‐115, 684, 279 between exon3 and exon4) using the fragments interface on the catRAPID website, peptides 114–165 belong to TDP‐43 RRM1, 201–252 peptides belong to TDP‐43 RRM2. The representative image shows the forecast on the binding site between TDP‐43 and *GAP43* pre‐mRNA. The Interaction Score showing the strength of catRAPID interaction propensities upon the *GAP43* RNA fragment sequence. Red shading in the heatmap indicates the interaction scores for individual amino acid and nucleotide pairs. B) Schematic diagram of the *GAP43* minigene and the primer pairs (arrows) for RT‐PCR. The splicing products of *GAP43* CE in shTDP‐43 HEK‐293T cells were analyzed by RT‐PCR, and quantitated in a violin plot (n = 3). Data are mean ± S.D.; *P* value for shTDP‐43 versus shCTR (Unpaired two‐tailed Student's *t* test). C) Schematic diagram of the primers (arrows) of GAP43 in RNA IP (RIP) for RT‐PCR, and quantified (n = 3). Data are mean ± S.D.; *P* value for TDP‐43 versus IgG (Unpaired two‐tailed Student's *t* test). Western blotting analysis of TDP‐43 in the immunoprecipitates of RIP. Arrowhead, IgH, heavy chain of IgG. D) Schematic diagram of *GAP43 4a1* intron reporters, and the Western blotting analysis of GFP in HEK‐293T cells transfected with pEGFP‐*4a1* and HA‐TDP‐43 (n = 3), and quantification. Data are mean ± S.D.; *P* value for pEGFP‐*4a1* with HA‐TDP‐43 or shTDP‐43 versus pEGFP‐*4a1* (One‐Way ANOVA, Tukey's multiple comparison). E) The splicing products of *GAP43 4a1* in HEK‐293T cells transfected with pEGFP‐*4a1* and TDP‐43 mutants were analyzed by RT‐PCR, and quantitated (n = 3). Data are mean ± S.D.; *P* value for pEGFP‐*4a1* and TDP‐43 mutants versus pCI‐neo (One‐Way ANOVA, Tukey's multiple comparison). F) EMSA assay showing that TDP‐43 protein had a higher affinity to bind *GAP43 4a1* RNA probe. G) Ideograph of the splicing of *GAP43* CE when TDP‐43 is depleted.

To identify the TDP‐43 binding sites within *GAP43* pre‐mRNA between exons 3 and 4, we used in silico prediction with catRAPID.^[^ ^34^ ^]^ The analysis revealed that the RRM1 of TDP‐43 exhibited the highest binding affinity for the *GAP43* pre‐mRNA region spanning chr3:115,676,611–115,578,800 (**Figure** **3**A).

Experimental validation involved constructing a *GAP43* minigene containing the predicted binding region and transfecting it into HEK‐293T cells. Cryptic exon *4a1* inclusion increased significantly in both the control and shTDP‐43 groups (Figure 3B). This unexpected increase in the control group suggests that essential repressor elements, present in the full‐length *GAP43* intron (~43,000 bp), were lost in the minigene construct, which retained only 2,190 bp. Assessing direct interaction between TDP‐43 and *GAP43* pre‐mRNA, we performed endogenous RNA immunoprecipitation (RIP) in M17 cells using three primer pairs (Rip‐F1/Rip‐R1, Rip‐F2/Rip‐R2, and Rip‐F3/Rip‐R3). While Rip‐F1/Rip‐R1 and Rip‐F2/Rip‐R2 amplified bands in both the input and IgG control groups, no enrichment was detected in the TDP‐43 immunoprecipitation (IP) group (Figure S5A, Supporting Information). However, the Rip‐F3/Rip‐R3 primers produced a distinct 160 bp band in both the input and TDP‐43 IP groups (Figure 3C), confirming that TDP‐43 binds to this region of *GAP43* pre‐mRNA.

Further investigation of the direct interaction between TDP‐43 and cryptic exon *4a1* involved engineering a *GAP43 4a1* reporter system by inserting *4a1* into the pEGFP vector between amino acids 126 and 127 of EGFP. Flanking sequences (AG and GU splice donor‐acceptor motifs) prevented alternative TDP‐43 binding sites. Co‐transfection of the reporter construct with either full‐length TDP‐43 (TDP‐43^FL^) or shTDP‐43 plasmids into HEK‐293T cells, followed by Western blotting analysis, revealed a significant increase in EGFP levels in the TDP‐43^FL^ group and a reduction in the shTDP‐43 group (Figure 3D), supporting the hypothesis that TDP‐43 directly binds to *GAP43 4a1* and represses its inclusion. To pinpoint the specific RRM responsible for binding *4a1*, we transfected HEK‐293T cells with the *GAP43 4a1* reporter construct alongside TDP‐43 mutants with inactivated (TDP‐43^F147/149L^ and TDP‐43^F227/229L^), or deleted (TDP‐43^ΔRRM1^ and TDP‐43^ΔRRM2^) RRMs mutants. Splicing repression of *GAP43* was significantly impaired in the TDP‐43^F147/149L^ and TDP‐43^ΔRRM1^ groups, whereas no significant change occurred in the TDP‐43^F227/229L^ and TDP‐43^ΔRRM2^ groups (Figure 3E). These results indicate that RRM1, but not RRM2, is essential for TDP‐43‐mediated repression of *GAP43 4a1* inclusion. Electrophoretic mobility shift assays (EMSA) using a synthetic *GAP43 4a1* probe further confirmed the direct interaction between TDP‐43 and *4a1* (Figure 3F). Collectively, these findings demonstrate that TDP‐43 binds *GAP43* pre‐mRNA via RRM1 and represses the splicing of cryptic exon *4a1* (Figure 3G).

### 2.4 TDP‐43 Oligomerization and Phosphorylation Modulate *GAP43* Cryptic Exon Inclusion

**Figure 4:**
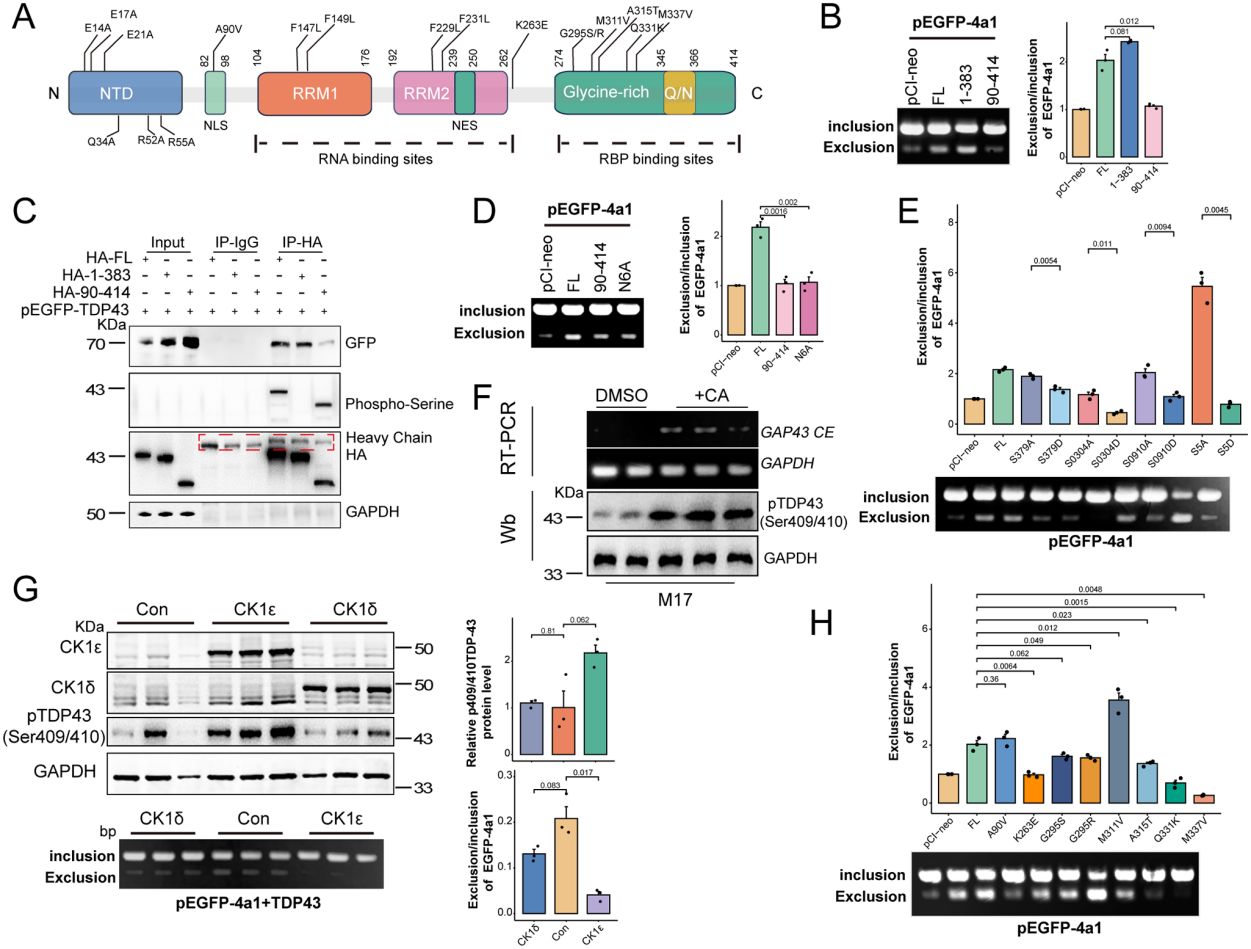
Oligomerization and phosphorylation of TDP‐43 influence its binding to *GAP43 4a1*. A) Schematic diagram of TDP‐43 with an N‐terminal domain (NTD), two RRM domains, a nuclear export signal (NES), a nuclear localization signal (NLS), a prion‐like disordered C‐terminal domain with glutamine/asparagine (Q/N)‐ and Glycine‐rich regions with ALS‐/FTD‐related mutations at C‐terminus. B, D, E, H) The splicing products of *GAP43 4a1* in HEK‐293T cells transfected with pEGFP‐*4a1* and TDP‐43 mutants were analyzed by RT‐PCR, and quantitated (n = 3). Data are mean ± S.D.; *P* value for pEGFP‐*4a1* and TDP‐43 mutants versus pCI‐neo (One‐Way ANOVA, Tukey's multiple comparison). C) Western blotting analysis of GFP and phospho‐Serine in HEK‐293T cells transfected with pEGFP‐TDP‐43 and full length (TDP‐43^FL^) or truncated (TDP‐43^1‐383^, TDP‐43^90‐414^) TDP‐43 with HA tag. F) The splicing products of *GAP43* CE in M17 cells treated with Calyculin A (CA) were analyzed by RT‐PCR (upper panel). Western blotting analysis of pTDP‐43 (Ser409/410) in CA treated M17 cells (lower panel). G) Western blotting analysis of pTDP‐43 (Ser409/410) in HEK‐293T cells transfected with CK1ε or CK1δ (left panel), or the splicing products of *GAP43 4a1* in HEK‐293T cells transfected with pEGFP‐*4a1* and CK1ε or CK1δ were analyzed by RT‐PCR (right panel), and quantitated (n = 3). Data are mean ± S.D.; *P* value for CK1 versus control (One‐Way ANOVA, Tukey's multiple comparison).

TDP‐43 consists of an N‐terminal domain involved in self‐oligomerization, two RRMs, and a glycine‐rich C‐terminal region containing multiple phosphorylation sites and ALS‐/FTD‐associated mutations (**Figure** **4**A).^[^ ^35^ ^]^ Hyperphosphorylated TDP‐43 inclusions are a common feature of various neurodegenerative diseases.^[^ ^36^, ^37^ ^]^ To investigate the role of TDP‐43 domains in *GAP43* splicing regulation, we co‐transfected truncated TDP‐43 constructs—TDP‐43^1‐383^ (lacking the C‐terminus) or TDP‐43^90‐414^ (lacking the N‐terminus)—with pEGFP‐*4a1* into HEK‐293T cells and performed RT‐PCR. Compared to full‐length TDP‐43 (TDP‐43^FL^), cryptic exon *4a1* exclusion was enhanced in the TDP‐43^1‐383^ group and reduced in the TDP‐43^90‐414^ group (Figure 4B), suggesting that oligomerization and phosphorylation influence TDP‐43′s interaction with *GAP43 4a1*. To further confirm this, we transfected HA‐tagged TDP‐43^1‐383^ or TDP‐43^90‐414^ alongside GFP‐TDP‐43^FL^ into HEK‐293T cells, followed by immunoprecipitation with anti‐HA antibody and Western blotting analysis. Phosphorylation was significantly reduced in the TDP‐43^1‐383^ group, indicating that TDP‐43 phosphorylation is critical for its interaction with *GAP43 4a1*. Assessing oligomerization through GFP immunoprecipitation, we found a reduction in GFP levels in the TDP‐43^90‐414^ group, suggesting that N‐terminal truncation impairs TDP‐43 aggregation (Figure 4C). Given that the 6A mutation in the N‐terminal domain of TDP‐43 affects oligomerization,^[^ ^38^ ^]^ we co‐transfected TDP‐43^N6A^ with pEGFP‐*4a1* and performed to RT‐PCR. No change in *4a1* exclusion was observed (Figure 4D), further supporting the role of TDP‐43 oligomerization in regulating *GAP43* splicing.

To determine whether TDP‐43 phosphorylation modulates its interaction with *GAP43 4a1*, we co‐transfected phosphor‐blocking (TDP‐43^S379A^, TDP‐43^S403/404A^, TDP‐43^S409/410A^, TDP‐43^S5A^) or phosphor‐mimicking (TDP‐43^S379D^, TDP‐43^S403/404D^, TDP‐43^S409/410D^, TDP‐43^S5D^) TDP‐43 mutants with pEGFP‐*4a1* and analyzed splicing by RT‐PCR. Compared to the phosphor‐blocking groups, *GAP43 4a1* exclusion was significantly reduced in the phosphor‐mimicking groups. Notably, exclusion was further enhanced in TDP‐43^S5A^ compared to TDP‐43^FL^, with the most pronounced difference between the TDP‐43^S5A^ and TDP‐43^S5D^ groups (Figure 4E).

TDP‐43 is dephosphorylated by protein phosphatase 1 (PP1).^[^ ^39^ ^]^ To assess whether PP1 inhibition affects *GAP43* splicing, we transfected *GAP43* minigene into HEK‐293T cells, treated them with Calyculin A (CA), a selective PP1 inhibitor, and performed to RT‐PCR. CA treatment markedly increased *GAP43* cryptic splicing (Figure S5B, Supporting Information). Similarly, treating M17 cells with CA significantly upregulated endogenous *GAP43 4a1* levels (Figure  4F).

Several kinases, including casein kinase (CK) 1ε and CK1δ, phosphorylate TDP‐43.^[^ ^40^, ^41^ ^]^ Western blotting analysis following CK1ε or CK1δ transfection into HEK‐293T cells confirmed TDP‐43 phosphorylation, with CK1ε exhibiting stronger effects (Figure 4G). Co‐transfection of CK1ε or CK1δ with pEGFP‐*4a1* revealed that both kinases significantly reduced *4a1* exclusion (Figure 4G). These findings indicate that TDP‐43 phosphorylation enhances *GAP43* cryptic exon inclusion, while dephosphorylation promotes *4a1* exclusion.

To assess whether ALS‐/FTD‐associated TDP‐43 mutations influence *GAP43* splicing, we co‐transfected pEGFP‐*4a1* with various mutant TDP‐43 constructs, including TDP‐43^A90V^, TDP‐43^K263E^, TDP‐43^G295S^, TDP‐43^G295R^, TDP‐43^M311V^, TDP‐43^A315T^, TDP‐43^Q311K^, and TDP‐43^M337V^, and analyzed RT‐PCR results. Compared to TDP‐43^FL^, *GAP43 4a1* exclusion was decreased in the TDP‐43^K263E^, TDP‐43^Q311K^, and TDP‐43^M337V^ groups, while increased in the TDP‐43^M311V^ group (Figure 4H). In summary, these findings suggest that TDP‐43 oligomerization is critical for the repression of *GAP43* mis‐splicing, whereas its hyperphosphorylation may result in a functional impairment that compromises its ability to regulate *GAP43* splicing.

### 2.5 Phosphorylation and Oligomerization of TDP‐43 Regulate *GAP43* Cryptic Splicing Independently of Subcellular Localization

**Figure 5:**
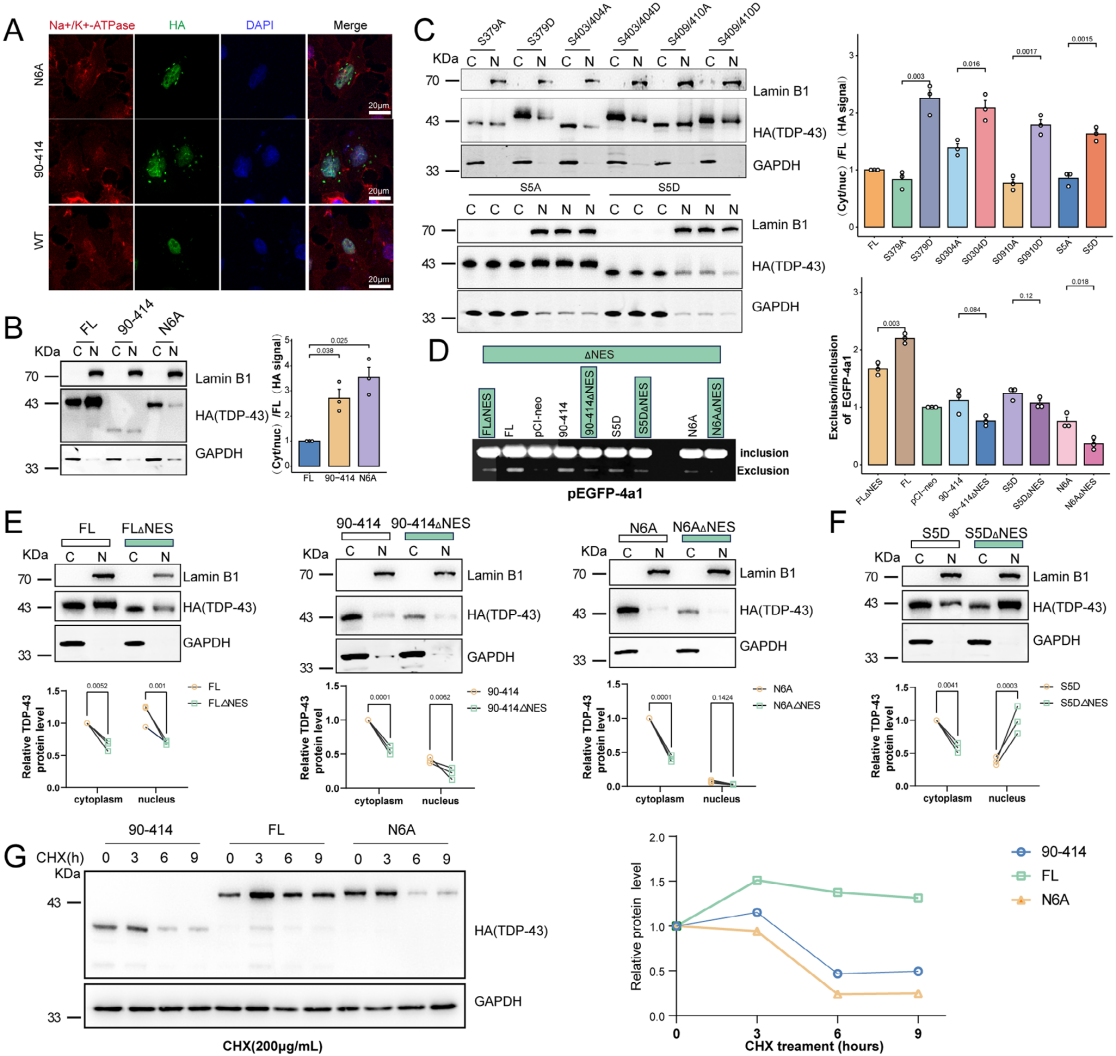
Phosphorylation and oligomerization of TDP‐43 affect the cryptic splicing of *GAP43*, but the effect is not medicated by altering cellular localization of TDP‐43. A) Representative images of the cytomembrane (Na^+^/K^+^‐ATPase, red) or nucleus (DAPI, blue) staining of TDP‐43 in HeLa cells transfected with TDP‐43^FL^, TDP‐43^90‐414^ or TDP‐43^N6A^. Scale bar, 100 µm. B,C) Western blotting analysis of TDP‐43 (HA) in the cytoplasm (C) or nucleus (N) fractionation in HeLa cells transfected with mutations or truncations of TDP‐43 (TDP‐43^FL^; TDP‐43^90‐414^; TDP‐43^N6A^; TDP‐43^S379A^; TDP‐43^S379D^; TDP‐43^S403/404A^; TDP‐43^S403/404D^; TDP‐43^S409/410A^; TDP‐43^S409/410D^; TDP‐43^S5A^; TDP‐43^S5D^), and quantification (n = 3). Data are mean ± S.D.; *P* value for TDP‐43 truncation or mutation versus full length (FL) (One‐Way ANOVA, Tukey's multiple comparison). D) The splicing products of GAP43 *4a1* in HEK‐293T cells transfected with pEGFP‐*4a1* and TDP‐43^FL^, TDP‐43^90‐414^, TDP‐43^S5D^, TDP‐43^N6A^ or the ΔNES mutants were analyzed by RT‐PCR, and quantitated (n = 3). Data are mean ± S.D.; *P* value for TDP‐43 mutants versus pCI‐neo (One‐Way ANOVA, Tukey's multiple comparison). E,F), Western blotting analysis of TDP‐43 (HA) in the cytoplasm (C) or nucleus (N) fractionation in HeLa cells transfected with mutations or truncations of TDP‐43 (TDP‐43^FL^; TDP‐43^FLΔNES^; TDP‐43^90‐414^; TDP‐43^90‐414ΔNES^; TDP‐43^N6A^; TDP‐43^N6AΔNES^; TDP‐43^S5D^; TDP‐43^S5DΔNES^), and quantification (n = 3). Data are mean ± S.D.; *P* value for normal versus ΔNES mutants (Two‐Way ANOVA, Sidak's multiple comparison). G), Western blotting analysis of TDP‐43 (anti‐HA) in HEK‐293T cells transfected with TDP‐43^FL^, TDP‐43^90‐414^ or TDP‐43^N6A^ and treated with CHX (200 µg mL^−1^) for 0, 3, 6, 9 h, and quantiification (TDP‐43^FL^, green; TDP‐43^90‐414^, purple; and TDP‐43^N6A^, orange).

Phosphorylation and oligomerization of TDP‐43 have been reported to alter its intracellular localization.^[^ ^38^, ^42^ ^]^ To examine this, we transfected HeLa cells with TDP‐43^FL^, TDP‐43^90‐414^, and TDP‐43^N6A^ and performed immunofluorescence staining using anti‐HA and anti‐Na⁺/K⁺‐ATPase antibodies (**Figure** **5**A). Compared to TDP‐43^FL^, cytoplasmic aggregations were more pronounced in the TDP‐43^90‐414^ and TDP‐43^N6A^ mutants (Figure 5A). Further nucleocytoplasmic fractionation in HEK‐293T cells revealed a significantly increased cytoplasm‐to‐nucleus ratio in these mutants compared to TDP‐43^FL^ (Figure 5B), indicating that TDP‐43 oligomerization may facilitate nuclear retention, while its disruption promotes cytoplasmic mislocalization.

Next, we investigated whether phosphorylation affects TDP‐43 nuclear translocation. Phosphor‐blocking (TDP‐43^S379A^, TDP‐43^S403/404A^, TDP‐43^S409/410A^, TDP‐43^S5A^) or phosphor‐mimicking (TDP‐43^S379D^, TDP‐43^S403/404D^, TDP‐43^S409/410D^, TDP‐43^S5D^) mutants were transfected into HeLa cells, followed by immunofluorescence staining (Figure S5C, Supporting Information). Cytoplasmic TDP‐43 aggregations were observed in the phosphor‐mimicking groups, while phosphor‐blocking mutants TDP‐43^S409/410A^ and TDP‐43^S5A^ exhibited perinuclear cytoplasmic inclusions. Western blotting analysis of nucleocytoplasmic fractionation in HEK‐293T cells showed a significant increase in the cytoplasm‐to‐nucleus ratio of phosphor‐mimicking mutants (Figure 5C). These findings further support that TDP‐43 phosphorylation promotes its cytoplasmic localization, consistent with previous studies.^[^ ^38^, ^42^ ^]^

To investigate whether TDP‐43 oligomerization and phosphorylation regulate *GAP43* cryptic splicing through changes in its nucleocytoplasmic localization, we generated nuclear export signal (NES)‐deleted constructs to enhance nuclear accumulation of TDP‐43, including TDP‐43^FLΔNES^, TDP‐43^90‐414ΔNES^, TDP‐43^N6AΔNES^, and TDP‐43^S5DΔNES^. These constructs were co‐transfected with pEGFP‐*4a1* into HEK‐293T cells, followed by RT‐PCR analysis. The results showed that deletion of the NES did not enhance the ability of these TDP‐43 variants to suppress *GAP43* cryptic exon inclusion (Figure 5D). To further understand this phenomenon, we assessed nucleocytoplasmic fractionation and Western blotting of HEK‐293T cells overexpressing NES‐deleted constructs. Compared with TDP‐43^S5D^, nuclear retention was significantly increased in TDP‐43^S5DΔNES^ (Figure 5F). This suggests that the loss of TDP‐43′s ability to repress *GAP43* mis‐splicing due to hyperphosphorylation is not dependent on its mis‐localization to the cytoplasm. However, no corresponding nuclear accumulation was observed for TDP‐43^90‐414ΔNES^ and TDP‐43^N6AΔNES^ (Figure 5E). We speculate that these mutants may undergo rapid nuclear degradation, impairing their oligomerization function. TDP‐43 oligomerization is known to stabilize the protein within the nucleus.^[^ ^38^ ^]^ To examine whether oligomerization affects TDP‐43 stability, we overexpressed TDP‐43^FL^, TDP‐43^90‐414^, and TDP‐43^N6A^ in HEK‐293T cells and treated them with CHX, a protein synthesis inhibitor, followed by Western blotting analysis. Compared to TDP‐43^FL^, TDP‐43 was degraded more rapidly in the TDP‐43^90‐414^ and TDP‐43^N6A^ groups (Figure 5G), further supporting the role of oligomerization in TDP‐43 nuclear stability. These results suggest that the nuclear retention of TDP‐43 alone does not explain its impact on *GAP43* cryptic splicing.

To determine whether ALS‐/FTD‐associated TDP‐43 mutations influence intracellular localization, we overexpressed TDP‐43^A90V^, TDP‐43^K263E^, TDP‐43^G295S^, TDP‐43^G295R^, TDP‐43^M311V^, TDP‐43^A315T^, TDP‐43^Q331K^, and TDP‐43^M337V^ in HeLa cells, followed by immunofluorescence staining and nucleocytoplasmic fractionation (Figure S5D, Supporting Information). Significant cytoplasmic translocation of TDP‐43 was observed in the TDP‐43^K263E^, TDP‐43^A315T^, TDP‐43^Q331K^, and TDP‐43^M337V^ groups. Additionally, TDP‐43^K263E^ and TDP‐43^A315T^ exhibited diffuse cytoplasmic expression, while TDP‐43^Q331K^ and TDP‐43^M337V^ formed cytoplasmic inclusions. Together, these results suggest that the effect of TDP‐43 phosphorylation and oligomerization on *GAP43* cryptic splicing is not mediated by alterations in cellular localization. Instead, oligomerization appears to be essential for TDP‐43 stability and function, whereas phosphorylation promotes its cytoplasmic mislocalization, potentially contributing to neurodegenerative disease pathology.

### 2.6 *GAP43* CE *4a1* is Up‐Regulated in AD Brains with pTDP‐43 Pathology

**Figure 6:**
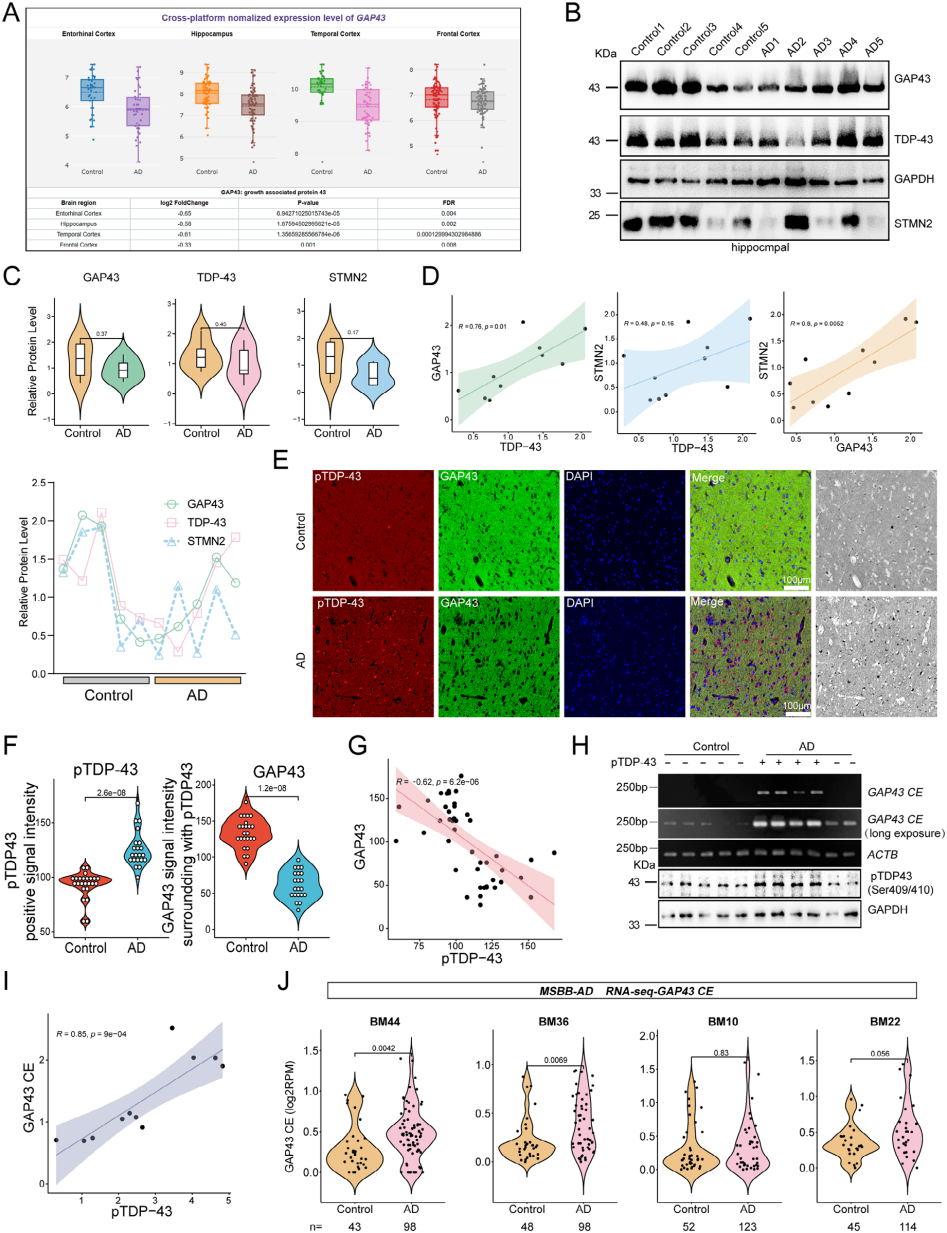
Cryptic splicing of *GAP43* mRNA is a hallmark of AD patients with pTDP‐43 pathology. A) *GAP43* expression in the normal population and AD patients generated from the AlzData datasets. B–D) Western blotting analysis of GAP43 and TDP‐43 in brain extracts from AD patients and age‐matched controls (B), and the quantification of GAP43 (green), TDP‐43 (red), and STMN2 (blue) protein level (n = 5) (C), and the Pearson's correlation of TDP‐43, STMN2 and GAP43 (n = 10) (D) Data are mean ± S.D.; *P* value for control versus AD patients; ns, no significance (C, Unpaired two‐tailed Student's *t* test. D, Simple Linear Regression Analysis). E‐G), Representative images of pTDP‐43 (red) and GAP43 (green) staining in temporal sections of normal control or AD patients (E) quantitated the signal intensity (F) and the Pearson's correlation of the signal intensity of GAP43 versus pTDP‐43 (G). Data are mean ± S.D.; *P* value for normal versus AD patients (F, Unpaired two‐tailed Student's *t* test. G, Simple Linear Regression Analysis). Scale bar, 100 µm. H,I) The splicing products of *GAP43* CE in control or AD brain extracts with/without pTDP‐43 were analyzed by RT‐PCR (H), and performed to Pearson's correlation of *GAP43* CE versus pTDP‐43 (n = 11) (I). J) The level of cryptic exon of *GAP43* in post‐mortem brain tissues from normal controls (Braak stages 0–VI). BM10, frontal pole; BM22, superior temporal gyrus; BM36, parahippocampal gyrus; and BM44, inferior frontal gyrus. Data are mean ± S.D.; *P* value for control versus AD patients (Wilcoxon rank sun test).

Phosphorylated TDP‐43 (pTDP‐43) is observed in approximately two‐thirds of AD brains.^1,9^ In this study, we demonstrated that TDP‐43 phosphorylation disrupts its binding to *GAP43* cryptic exon *4a1* and induces aberrant *GAP43* transcripts (Figure 4E). To determine whether GAP43 expression is reduced in AD brains, we analyzed the AlzData dataset and found that full‐length *STMN2* and *GAP43* transcripts were significantly decreased in the frontal cortex, temporal cortex, hippocampus, and entorhinal cortex of AD patients compared to age‐matched controls (**Figure** **6**A).

Western blotting analysis of AD brain homogenates revealed a downward trend in GAP43, STMN2, and TDP‐43 protein levels; however, the differences were not statistically significant, most likely due to the small sample sizes and high data variability (Figure 6B,C). Notably, the expression curves of GAP43, STMN2, and TDP‐43 showed a strong concordance (Figure 6C). Correlation analysis indicated that TDP‐43 levels were more strongly associated with GAP43 expression than with STMN2 (Figure 6D). To further examine the relationship between pTDP‐43 and GAP43, we performed immunofluorescence staining using anti‐pTDP‐43 and anti‐GAP43 antibodies on temporal lobe sections from AD patients with pTDP‐43 pathology, obtained from the National Health and Disease Human Brain Tissue Resource Center (Figure 6E and Figure S6, Supporting Information). Quantification of fluorescence intensity revealed a significant increase in pTDP‐43 and a corresponding decrease in GAP43 expression in AD brains compared to controls (Figure 6F). Additionally, a negative correlation was observed between pTDP‐43 and GAP43 levels (Figure 6G).

To assess whether *GAP43* cryptic exon *4a1* inclusion is associated with pTDP‐43 pathology, we extracted RNA from temporal lobe tissues of AD patients with or without pTDP‐43 and analyzed *4a1* levels by RT‐PCR. *GAP43 4a1* inclusion was significantly elevated only in pTDP‐43 positive AD patients (Figure 6H,I), with a positive correlation between pTDP‐43 levels and *GAP43* cryptic exon *4a1* splicing. More importantly, we further analyzed the expression of *GAP43* cryptic exon *4a1* transcripts in bulk RNA‐seq data from post‐mortem brain tissues of AD patients and brain region‐matched controls from the Mount Sinai/JJ Peters VA Medical Center Brain Bank (MSBB‐AD). We found that *GAP43* cryptic exon *4a1* was significantly upregulated in the parahippocampal gyrus (BM36) and inferior frontal gyrus (BM44) (Figure 6J), whereas no significant changes were observed in the frontal pole (BM10) or superior temporal gyrus (BM22) (Figure 6J). Together, these findings suggest that *GAP43* cryptic exon *4a1* may serve as a potential biomarker for AD patients with pTDP‐43 pathology.

### 2.7 GAP43 Rescues the Defect of Axon Growth Cone Development Induced by TDP‐43 Deficiency

**Figure 7:**
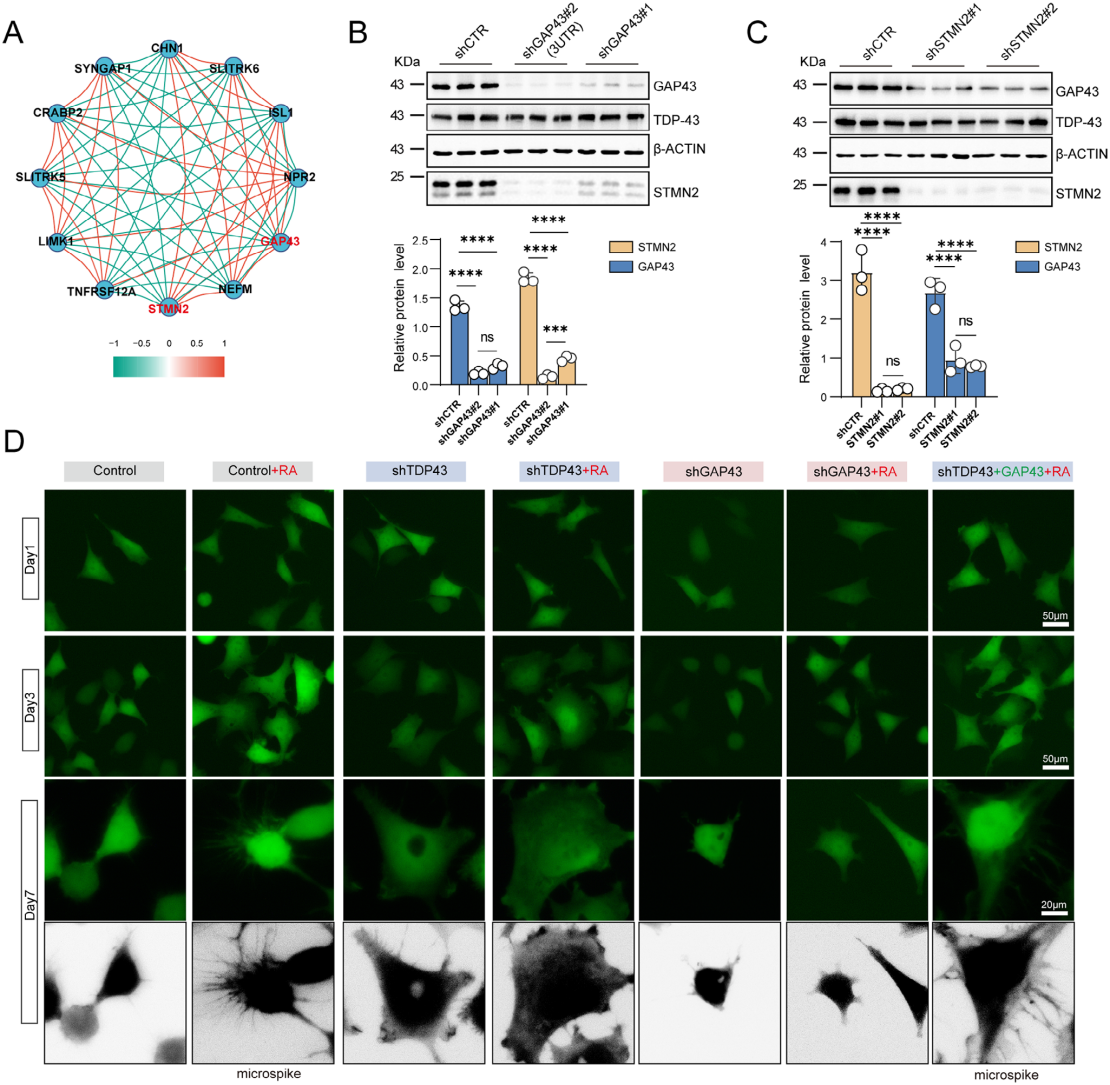
Restoration of GAP43 attenuates RA‐induced impairment of axonal growth cone development after TDP‐43 depletion. A) Correlation network plot showing the Pearson's correlation between highly variable synapse‐associated genes in TDP‐43 knockdown M17 cells. B,C) Western blotting analysis of GAP43 and STMN2 in HEK‐293T cells transfected with shGAP43#1, shGAP43#2 (3′UTR) (B), or shSTMN2#1, shSTMN2#2 (C), and quantification (n = 3). UTR, untranslated region. Data are mean ± S.D.; ^***^ *P* < 0.001; ^****^ *P* < 0.0001 for knockdown versus control; ns, no significance (Two‐Way ANOVA, Sidak's multiple comparison). D) Representative images of the morphology of DOX‐inducible shTDP‐43 stable M17 cells treated with doxycycline, shGAP43 or doxycycline with overexpression GAP43 lentiviruses on day 1, day 3 (Scale bar, 50 µm), and day 7 (Scale bar, 20 µm) after RA (10 µm) or control treatment.

Our study revealed a positive correlation between GAP43 and STMN2 expression in AD brains (Figure 6D). We then analyzed the correlation of highly variable synapse‐associated genes in RNA‐seq data from TDP‐43 knockdown M17 cells and further confirmed the positive association between *GAP43* and *STMN2* (**Figure** **7**A). To validate this relationship at the protein level, we knocked down GAP43 in M17 cells and assessed STMN2 expression via Western blotting. STMN2 levels were significantly reduced following GAP43 knockdown (Figure 7B). Likewise, GAP43 expression was markedly decreased upon STMN2 knockdown (Figure 7C).

Next, we performed rescue experiments by overexpressing GAP43 in M17 cells under conditions of either GAP43 or TDP‐43 knockdown. GAP43 overexpression restored STMN2 expression in cells with GAP43 knockdown (Figure S7A, Supporting Information); however, under DOX‐induced TDP‐43 knockdown, GAP43 overexpression failed to rescue STMN2 levels (Figure S7B, Supporting Information).

To investigate the effect of TDP‐43 depletion‐induced GAP43 downregulation on neuronal differentiation, we treated M17 cells with RA to induce differentiation.^[^ ^43^ ^]^ Between days 1 and 7 of RA treatment, growth cones and filopodia emerged. However, in DOX‐induced TDP‐43 knockdown M17 cells treated with RA, no growth cones or filopodia were observed by day 7 (Figure 7D). Notably, these cells displayed a striking morphological change characterized by swollen cell bodies.

Meanwhile, RA treatment significantly upregulated GAP43 expression in control cells, whereas STMN2 expression remained unchanged in the DOX‐induced group (Figure S8, Supporting Information), suggesting a critical role for GAP43 in the differentiation process.

To further evaluate the function of GAP43, we infected M17 cells with Lenti/shGAP43#2 and treated them with RA for 7 days. GAP43 knockdown did not result in growth cone or filopodia formation, nor did it induce cell body swelling (Figure 7D), supporting the notion that GAP43 is essential for proper growth cone development during differentiation.

Finally, to assess whether exogenous GAP43 could rescue growth cone defects caused by TDP‐43 loss, we overexpressed GAP43 in TDP‐43‐depleted M17 cells. This restored growth cone formation, although cell body swelling persisted (Figure 7D). These findings strongly suggest that loss of GAP43 is a key downstream consequence of TDP‐43 dysfunction, contributing to impaired growth cone formation.

### 2.8 Impaired Axonal Regeneration on TDP‐43 Loss in Human iPSC‐derived Motor Neurons is Alleviated by GAP43 Restoration

Previous studies have demonstrated that knockdown of TDP‐43 impairs axonal regeneration in human iPSC‐derived motor neurons (iPSC‐MNs).^[^ ^9^, ^10^ ^]^ Given that GAP43 is a well‐established regulator of axonal outgrowth,^[^ ^14^, ^15^, ^16^ ^]^ we investigated whether the axonal regeneration deficits observed upon TDP‐43 depletion are directly attributable to reduced GAP43 expression.

**Figure 8:**
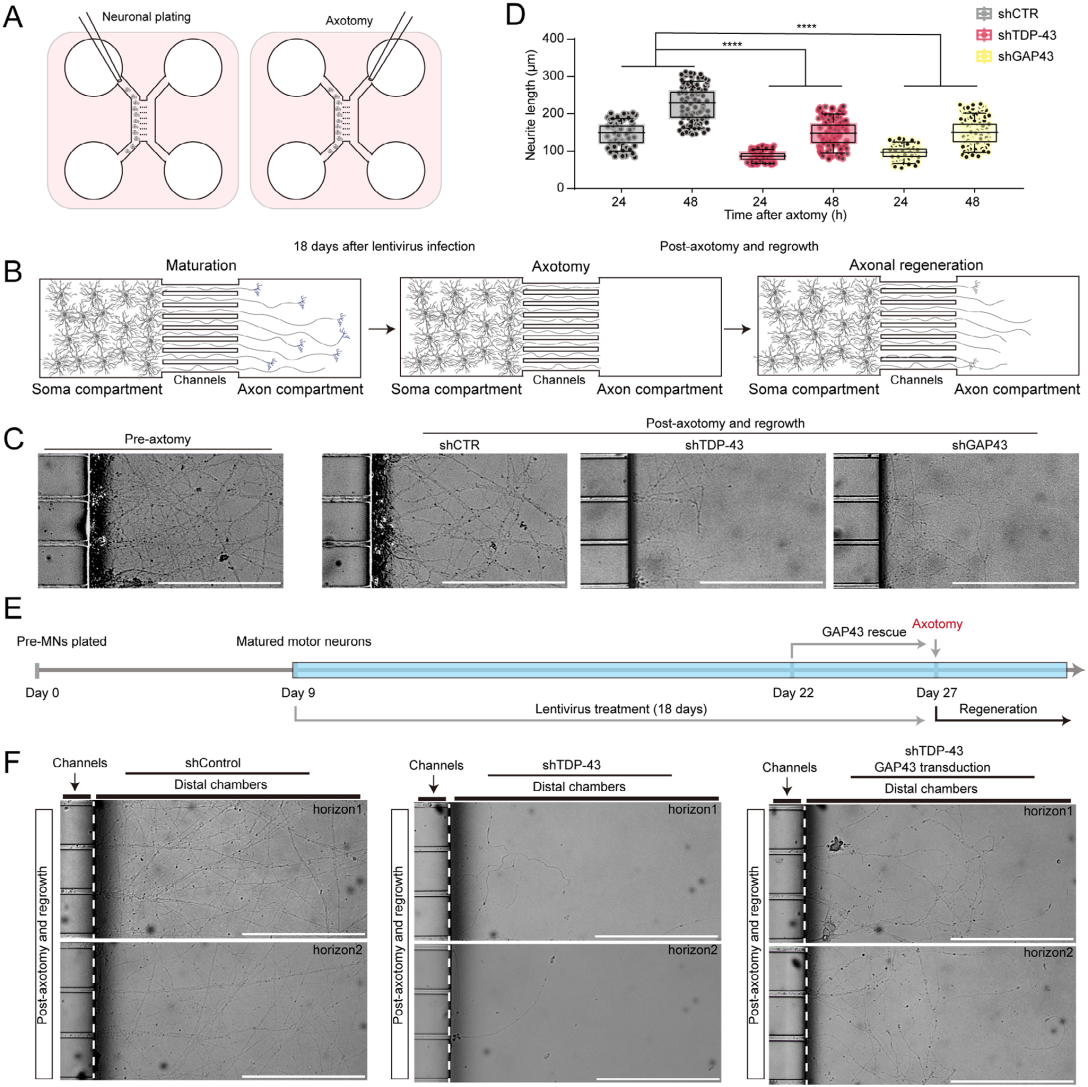
Restoration of GAP43 rescues axonal regeneration impairment after TDP‐43 depletion in iPSC‐MNs. A) Schematic of axotomy assay in iPSC‐MNs in microfluidics chambers. iPSC‐MNs, iPSC‐derived motor neurons. B) Representative micrographs of iPSC‐MNs in microfluidics chambers pre‐ and post‐ axotomy with lentiviruses of scramble, shTDP‐43 or shGAP43 (Scale bar, 150 µm). C) Measurements of neurite length in 24 and 48 h after axotomy. Individual neurites are displayed as dots along with mean ±SD (Unpaired *t*‐test, two‐sided, *P* value < 0.0001) (shCTR versus shTDP‐43, 24 h≤0.0001, 48 h≤0.0001) (shCTR versus shTDP‐43, 24 h≤0.0001, 48 h≤0.0001). Similar results were obtained in n = 3 devices from 2 independent experiments. D,E) Schematic illustration of motor neurons in microfluidic chambers before and after axotomy (E). Motor neuron precursors were plated at the somatic/proximal compartment, and axons reached into the distal/axonal compartment during a maturation period of another 9 days. Lentiviral infection treatment for 18 days before axotomy. Timeline of iPSC‐MNs plated, maturation, lentivirus treatment, and axotomy is shown (D). F) Representative micrographs of iPSC‐MNs in distal chambers in 48 h post‐axotomy with shCTR, shTDP‐43, or shTDP‐43+GAP43 lentivirus treatment.

To address this, motor neuron precursors were seeded into the proximal (somatic) chamber of a microfluidic device (**Figure** **8**A). Over a 9‐day maturation period, axons extended through 830‐µm‐long microgrooves into the distal compartment, which excludes cell bodies and permits only axonal growth (Figure 8B). Mature motor neurons were then treated with lentiviral vectors for 18 days to suppress the expression of either TDP‐43 or GAP43. Axons in the distal compartment were mechanically severed without disturbing the soma, and axonal regrowth was assessed by quantifying axon extension from the microgrooves at defined time points (Figure 8B–D).

In control neurons treated with a non‐targeting lentivirus, axonal regrowth was initiated within 24 h post‐injury. In contrast, knockdown of either TDP‐43 or GAP43 nearly abolished axonal regeneration (Figure 8C). Furthermore, neurons in both knockdown groups exhibited significantly reduced axon outgrowth over time compared to controls, indicating that reduced GAP43 expression increases neuronal vulnerability to axonal injury.

To determine whether restoring GAP43 expression could rescue the regeneration defects caused by TDP‐43 loss, we transduced motor neurons with a lentiviral vector overexpressing GAP43 following 13 days of TDP‐43 knockdown. Despite prolonged GAP43 suppression, its re‐expression nearly fully restored axonal regrowth after injury (Figure 8E,F), supporting a model in which GAP43 acts downstream of TDP‐43 and is sufficient to compensate for its loss in the context of axonal regeneration.

## 3 Discussion

TDP‐43 is increasingly recognized for its role in repressing cryptic exon inclusion during RNA splicing. Previous work has identified *STMN2* and *UNC13A* as prominent TDP‐43 targets,^[^ ^6^, ^7^, ^8^, ^9^, ^10^ ^]^ where cryptic exons are abnormally incorporated upon TDP‐43 loss or functional impairment. These mis‐splicing events are strongly implicated in ALS and FTD, and similar changes have been observed in AD brains exhibiting pathological pTDP‐43. However, additional cryptic splicing targets of TDP‐43 and their contributions to disease pathogenesis remain poorly understood.

In this study, we uncover *GAP43* as a novel cryptic splicing target regulated by TDP‐43. Our data show that TDP‐43 directly binds *GAP43* pre‐mRNA through RRM1, preventing the inclusion of cryptic exon *4a1* (Figure 3G). Functional impairment of TDP‐43—through depletion, phosphorylation, or disease‐related mutations—results in aberrant *GAP43 4a1* incorporation, triggering premature stop codons and NMD (Figure 4B–H). Furthermore, we found that restoring GAP43 expression in TDP‐43–depleted M17 cells and iPSC‐MNs was sufficient to rescue growth cone formation and axonal regeneration, respectively. Consequently, GAP43 protein levels decline, hindering growth cone formation and axonal regeneration in neuronal models, likely contributing to neurodegeneration (Figure 7D and Figure 8B–F). Notably, GAP43 also exhibits a mutually supportive relationship with STMN2, another TDP‐43 target essential for neuronal health, underscoring how disrupted splicing in these convergent pathways may amplify neuronal vulnerability (Figure 7A–C).

In AD brains presenting pTDP‐43 pathology, GAP43 expression is similarly reduced (Figure 6A,B). and cryptic exon *4a1* inclusion is elevated (Figure 6H,J), suggesting a shared mechanism of neuronal injury across ALS and AD. Taken together, these findings illustrate a broader regulatory network governed by TDP‐43, wherein cryptic splicing defects converge on crucial synaptic and axonal maintenance genes. Restoring TDP‐43 function or preventing cryptic exon integration may offer a therapeutic avenue for halting or reversing neuronal degeneration in TDP‐43 proteinopathies.

Despite these insights, our study has several limitations. First, although our findings highlight a direct regulatory link between TDP‐43 and GAP43, the cell and tissue models used may not fully recapitulate the complexity of human disease, particularly given the heterogeneity of ALS and AD. Second, while we demonstrated the mechanistic interplay between TDP‐43 phosphorylation, oligomerization, and *GAP43* cryptic splicing, the precise contribution of each post‐translational modification to other TDP‐43 targets remains incompletely understood. Third, the sample size for our human tissue analysis was relatively small, which may have reduced statistical power in detecting subtle protein‐level differences. Additionally, although our work suggests *GAP43* cryptic exon *4a1* as a potential biomarker, larger‐scale studies in diverse patient cohorts are needed to validate its diagnostic and prognostic utility.

Looking ahead, further research into TDP‐43‐associated cryptic splicing holds considerable promise for revealing new therapeutic strategies. Systematic identification and characterization of additional cryptic exons will help elucidate how TDP‐43 orchestrates broader neuronal gene networks. Advanced RNA‐targeting technologies—such as antisense oligonucleotides or small molecules designed to modulate splicing—could be harnessed to prevent cryptic exon inclusion in conditions where TDP‐43 is functionally compromised. Moreover, detailed studies on how TDP‐43 phosphorylation, oligomerization, and nuclear/cytoplasmic shuttling alter cryptic splicing outcomes may uncover specific intervention points. Ultimately, unraveling the complex landscape of TDP‐43–mediated RNA regulation could lead to precise, mechanism‐based therapies for a range of TDP‐43 proteinopathies.

## 4 Experimental Section

### Human Brain Tissues

Post‐mortem human brain tissue samples used in this study were obtained from the National Human Brain Tissue Resource Center for Health and Disease (China). Comprehensive demographic, clinical, and neuropathological information for each case was provided in Table S1 (Supporting Information). All tissue donations were carried out with informed consent obtained from either the donors themselves or their legally authorized representatives, in accordance with protocols approved by the Ethics Committee of Tongji Medical College, Huazhong University of Science and Technology (Approval No. [2019] IEC(S775)).

Clinical and neuropathological diagnoses were established based on standardized criteria, as previously described. Subjects included in the study were rigorously screened according to both clinical evaluations and neuropathological assessments to ensure diagnostic accuracy. The primary clinical diagnoses of the enrolled cases consisted of AD and AD with pTDP‐43 inclusions, which were confirmed through post‐mortem neuropathological examination.

### Plasmids and Viral Constructs

Plasmids encoding wild‐type and truncated TDP‐43 constructs with HA‐tag (pCI/TDP‐43, pCI/TDP‐43^1−383^, pCI/TDP‐43^90−414^), phosphor‐blocking (S379A, S403/404A, S409/410A, S5A), phosphor‐mimicking (S379D, S403/404D, S409/410D, S5D), RRM inactivated or deleted, and ΔNES mutants of TDP‐43 with HA‐tag, as well as HA‐tagged casein kinase 1 isoforms ε and δ (pCI/CK1ε and pCI/CK1δ), were generated as previously described.

To investigate the effects of ALS/FTD‐associated TDP‐43 mutations, site‐directed mutagenesis was performed using the inverse PCR method to introduce the following mutations: N6A, A90V, K263E, G295R, G295S, M311V, A315T, Q331K, and M337V. The presence of the intended mutations was confirmed by Sanger sequencing.

For RNA interference experiments, lentiviral short hairpin RNA (shRNA) constructs targeting TDP‐43 (shTDP‐43), GAP43 (shGAP43#1/2), and STMN2 (shSTMN2#1/2) were generated by cloning specific shRNA sequences into the pLL3.7 lentiviral vector (Addgene, Watertown, MA, USA). The target sequences for each shRNA were as follows:

TDP‐43 (human): 5′‐GCTCTAATTCTGGTGCAGCAA‐3′, TDP‐43 (mouse): 5′‐GTAGATGTCTTCATTCCCAAA‐3′, GAP43‐1 (human): 5′‐CGTGGACACATAACAAGGAAA‐3′, GAP43‐2 (human): 5′‐GTCAAACAGTGTGGCTTAAAC‐3′, STMN2‐1 (human): 5′‐CAATTGGCAGAGAAGAGGGAA‐3′, STMN2‐2 (human): 5′‐GTGAGGCTAATCTAGCTGCTA‐3′. All plasmid constructs and viral preparations were sequence‐verified before use in experiments.

### Cell Culture and Transfection

Human embryonic kidney 293T (HEK‐293T) cells and mouse neuroblastoma (N2a) cells were cultured in Dulbecco's Modified Eagle Medium (DMEM; Gibco) supplemented with 10% fetal bovine serum (FBS; Vazyme) and maintained at 37 °C in a humidified incubator with 5% CO₂. Human neuroblastoma M17 cells were maintained under similar conditions but using DMEM/F12 medium (Gibco) supplemented with 10% FBS.

Plasmid transfections were performed using Lipofectamine 2000 (Invitrogen) according to the manufacturer's protocol. For shRNA‐mediated knockdown experiments, cells were plated on day 0, transduced with lentiviral shRNA constructs on day 2, and subjected to a media change on day 3 to remove residual virus. Cells were harvested for subsequent analyses, including RT‐qPCR and Western blotting, on day 6.

### Stable Lentiviral Transduction and Selection

Human neuroblastoma M17 cells were transduced with the pLV3‐H1‐2O2‐MCS‐shTDP‐43‐EGFP‐TetR‐Puro lentiviral vector, which harbors a doxycycline‐inducible shRNA cassette targeting TDP‐43. Following transduction, cells were subjected to puromycin selection (2 µg mL^−1^) for two weeks to ensure the establishment of a stable knockdown cell population.

For doxycycline dose–response experiments, the TDP‐43‐knockdown M17 cell pool was plated as single cells in order to establish clonal populations, which were subsequently expanded and characterized for further analyses.

### iPSC Maintenance and Differentiation into iPSC‐Derived Neurons

Induced pluripotent stem cells (iPSCs) derived from normal human skin fibroblasts were utilized in this study. The detailed protocol for iPSC generation, maintenance, and differentiation is described in reference.

Briefly, iPSCs were cultured under standard feeder‐free conditions using mTeSR1 medium (STEMCELL Technologies) on Matrigel‐coated plates (Corning). Differentiation into iPSC‐derived cortical neurons (iNeurons) was induced following an established protocol, involving sequential exposure to neural induction factors and differentiation media to promote neuronal lineage commitment. Cells were maintained at 37 °C in a humidified 5% CO₂ incubator, with regular media changes to support neuronal maturation.

Further experimental details regarding differentiation efficiency and characterization assays are available in reference.

### iPSC and Motor Neurons Differentiation

Differentiation into iPSC‐derived motor neurons (iPSC‐MNs) was performed following an established protocol. Briefly, iPSCs were dissociated and transferred into ultra‐low attachment flasks (Corning) to form 3D spheroids in a medium composed of DMEM/F12 and Neurobasal (ThermoFisher Scientific), supplemented with N2 (Thermo Fisher) and B‐27 Xeno‐Free (ThermoFisher Scientific). Small molecules (LDN193189, SB‐431542, and CHIR99021) were added to promote neuronal progenitor patterning of the spheroids, followed by motor neuron induction using retinoic acid, a Smoothened (Smo) agonist, and DAPT. After 14 days, the neuronal spheroids were enzymatically dissociated with papain and DNase (Worthington Biochemical), and plated onto poly‐D‐lysine/laminin‐coated plates in Neurobasal medium (ThermoFisher Scientific) supplemented with neurotrophic factors including BDNF, GDNF, and CNTF (R&D Systems).

### Western Blotting

Total cellular protein was extracted using RIPA lysis buffer and protein concentrations were quantified using the Bradford assay (Beyotime). Equal amounts of total protein were mixed with Laemmli sample buffer, denatured by boiling at 95 °C for 5 min, and subsequently separated via sodium dodecyl sulfate–polyacrylamide gel electrophoresis (SDS‐PAGE). Proteins were then transferred onto polyvinylidene fluoride membranes (Millipore Sigma, #IPVH00010) using a semi‐dry transfer system.

Membranes were blocked in 5% fat‐free milk prepared in Tris‐buffered saline (TBS) for 1 h at room temperature to prevent nonspecific binding. Following blocking, membranes were incubated overnight at 4 °C with primary antibodies diluted in blocking buffer (detailed information on antibodies is provided in Table S2, Supporting Information). After primary antibody incubation, membranes were washed three times with TBST (TBS containing 0.1% Tween‐20) and then incubated with horseradish peroxidase ‐conjugated secondary antibodies (1:5000, ABclonal) for 2 h at room temperature.

Following incubation, membranes were washed with TBST, and protein bands were visualized using an enhanced chemiluminescence Western blotting substrate. Signal detection was performed using the ChemiScope 6100 imaging system (Clinx). Densitometric analysis of specific immunosignals was conducted using Multi Gauge software V3.0 (Fuji Film, Minato, Tokyo, Japan).

### Immunocytochemistry and Immunohistochemistry

For immunocytochemistry, HeLa and M17 cells were cultured on coverslips in 24‐well plates, transfected, and treated as described above. Similarly, for immunohistochemistry, post‐mortem AD brain sections were processed for staining.

Cells or brain tissue sections were fixed in 4% paraformaldehyde (PFA) in phosphate‐buffered saline (PBS) for 15 min at room temperature. Following fixation, samples were permeabilized with 0.5% Triton X‐100 (Biosharp, BS084) in PBS for 15–30 min and then blocked in 3% goat serum in PBST (PBS containing 0.1% Triton X‐100) for 1 h at room temperature to minimize nonspecific antibody binding.

Primary antibody incubation was performed overnight at 4 °C or for up to 48 h, using antibodies detailed in Table S2 (Supporting Information). After primary incubation, samples were washed with PBS and subsequently incubated with CY3‐ or FITC‐conjugated species‐matched secondary antibodies (1:300, ABclonal) at room temperature for 1–2 h. Following secondary antibody incubation, samples were washed with PBST, and cells were mounted onto glass slides using DAPI‐containing Mounting Medium (ZSGB‐Bio, ZLI9557) to counterstain nuclei.

Fluorescence signals were visualized using a Zeiss LSM800 laser confocal microscope, and images were acquired under identical settings to ensure consistency across experimental conditions.

### RT‐PCR and RT‐qPCR

Total cellular RNA was extracted from cultured cells and iPSC‐derived neurons using Trizol reagent (Takara), following the manufacturer's protocol. RNA purity and concentration were assessed using a NanoDrop spectrophotometer (Thermo Fisher Scientific) to ensure high‐quality RNA for downstream applications.

For cDNA synthesis, first‐strand complementary DNA (cDNA) was generated using oligo(dT)_18_ primers with the ABScript III reverse transcription kit (ABclonal, RK20429) according to the manufacturer's instructions.

Reverse transcription polymerase chain reaction (RT‐PCR) was carried out using 2× Phanta Max Master Mix (Dye Plus) (Vazyme, P525‐02) under standard cycling conditions. For quantitative real‐time PCR (qPCR), reactions were performed using Universal SYBR Green Fast qPCR mix (ABclonal, RK21203) on a CFX96 real‐time PCR detection system (Bio‐Rad). To enhance specificity in detecting *GAP43* cryptic exon inclusion, an optimized combination of primers and probes was employed. The specific sequences of probes and primers used for all reactions are listed in Table S3 (Supporting Information).

Each reaction was run in technical triplicates, and gene expression levels were normalized to ACTB (β‐actin) as an internal control. Relative quantification of target RNA was performed using the ^ΔΔ^Ct method to ensure accurate and reproducible results.

### RNA Sequencing

Total RNA was extracted from TDP‐43 knockdown M17 cells and corresponding control (shCTR/shTDP‐43) using the method described above. To ensure reproducibility and statistical robustness, three biological replicates were sequenced for each condition. RNA sequencing (RNA‐Seq) was performed by HUADA Genetics, generating 10 gigabases (Gb) of sequencing data per sample. The sequencing libraries were prepared following standard protocols, and paired‐end sequencing was conducted on an Illumina platform. For data analysis and visualization, RNA‐Seq read alignment, differential gene expression analysis, and cryptic exon detection were performed using established bioinformatics pipelines.

### IGV Visualization

RNA‐Seq data were processed and converted into BAM files following alignment and mapping to the human reference genome (hg38) using default parameters. Sequence alignment was performed using STAR or HISAT2, ensuring high mapping accuracy and proper handling of splice junctions.

For visualization, Integrative Genomics Viewer (IGV) was utilized to inspect read coverage, splicing patterns, and cryptic exon inclusion. BAM files, along with their corresponding index files (BAI), were loaded into IGV to examine read distribution, alternative splicing events, and TDP‐43‐associated cryptic splicing changes in the dataset.

### Splicing Reporter Assay

To investigate the splicing regulation of *GAP43*, minigene constructs were generated by amplifying the genomic region from chr3:115,676,611–115,578,800 from neuronal genomic DNA. This fragment, encompassing *GAP43* exons 3 and 4 along with the intervening intron, was cloned into the CMV 7.1 expression vector. Additionally, a pEGFP‐*4a1* construct was engineered by amplifying a fragment spanning chr3:115,677,724–115,677,883, which includes the cryptic exon *4a1* of *GAP43*, with artificially added AG and GU splice donor/acceptor sites at both ends. This fragment was inserted into the pEGFP vector between amino acids 126 and 127 to assess cryptic exon inclusion.

For transfection experiments, the indicated splicing reporter constructs (500 ng per well) were transfected into cultured cells using Lipofectamine 2000 (Invitrogen) according to the manufacturer's instructions. 48 h post‐transfection, total RNA was extracted and reverse‐transcribed into cDNA using the high‐capacity cDNA reverse transcription kit (ABclonal).

To analyze splicing outcomes, PCR amplification was performed using OneTaq 2× Master Mix with standard buffer (Vazyme) under the following cycling conditions: initial denaturation at 95 °C for 30 s, followed by 35 cycles of 95 °C for 15 s, 60 °C for 15 s, and 72 °C for 10 s, with a final extension at 72 °C for 5 min. PCR reactions were carried out using the M2‐96G Gradient Thermal Cycler (RWD). The resulting PCR products were separated via 2–3% TAE agarose gel electrophoresis and visualized using the ChemiDoc XRS+ imaging system (Bio‐Rad). The sequences of primers used for PCR amplification are listed in Table S3 (Supporting Information).

### RNA‐Binding Protein Immunoprecipitation (RIP) Assay

RNA‐binding protein immunoprecipitation (RIP) was performed using the RIP RNA‐Binding Protein Immunoprecipitation Kit (GENE CREATE, JKR23003) according to the manufacturer's protocol. To investigate the interaction between TDP‐43 and *GAP43* mRNA, endogenous TDP‐43 was immunoprecipitated from M17 cell lysates using an anti‐TDP‐43 antibody (ProteinTech, 10782‐2‐AP). A control rabbit IgG antibody (GENE CREATE, JKR23003) was used as a negative control to rule out nonspecific RNA‐protein interactions.

Following immunoprecipitation, RNA was extracted from the precipitated complexes and subjected to RT‐PCR to detect the presence of *GAP43 4a1* transcripts, confirming the association of TDP‐43 with cryptic exon *4a1*‐containing mRNA. The sequences of primers used for amplification are listed in Table S2 (Supporting Information).

### RNA Fluorescence In Situ Hybridization (RNA‐FISH)

To detect *GAP43 4a1* transcripts, digoxigenin (DIG)‐labeled RNA probes were used for fluorescence in situ hybridization (FISH). The assay was conducted using the Enhanced Sensitive ISH Detection Kit IV (CY3) (BOSTER, MK1033) according to the manufacturer's instructions.

### RNA Fluorescence In Situ Hybridization (RNA‐FISH)—Cell Culture and Fixation

HEK‐293T cells were cultured on polylysine‐coated coverslips under standard conditions (37 °C, 5% CO₂). After reaching appropriate confluency, cells were washed three times with 0.5 m PBS (pH 7.4) for 2 min each. Cells were then fixed with 4% PFA containing 1/1000 diethyl pyrocarbonate for 20–30 min at room temperature (RT). Following fixation, cells were thoroughly washed with distilled water, air‐dried, and stored at ‐20 °C for at least two weeks before processing.

### RNA Fluorescence In Situ Hybridization (RNA‐FISH)—mRNA Exposure and Pre‐Hybridization

To expose mRNA fragments, cells were treated with a digestion solution containing 3% citric acid and freshly diluted pepsin (1 mL of 3% citric acid + 2 drops of concentrated pepsin) and incubated at 37 °C for 45 s. The reaction was stopped by washing cells with 0.5 m PBS (three times, 5 min each) followed by a final rinse in distilled water.

For pre‐hybridization, wet hybridization cassettes were prepared by adding 20 ml of 20% glycerol to the bottom of a dry cassette to maintain humidity. Pre‐hybridization solution (20 µL per well) was applied to the samples, and incubation was performed in a thermostat‐controlled chamber at 37–40 °C for 2–4 h. Excess liquid was aspirated without additional washing before hybridization.

### RNA Fluorescence In Situ Hybridization (RNA‐FISH)—Hybridization and Stringency Washes

Hybridization was carried out by diluting DIG‐labeled oligonucleotide probes in hybridization buffer to a final concentration of 2 µg mL^−1^. 20 µL of the hybridization solution was added to each well, and samples were incubated overnight at 37–40 °C. Post‐hybridization washes were performed under stringent conditions as follows: twice in 2× SSC at 30–37 °C for 5 min each, once in 0.5× SSC for 15 min, and once in 0.2× SSC for 15 min. Signal detection and fluorescence imaging were subsequently carried out.After hybridization and washing, sections were treated with a sealing solution at 37 °C for 30 min, followed by incubation with biotinylated mouse anti‐digoxin antibody at 37 °C for 60 min or RT for 2 h. After washing with 0.5 m PBS (four times, 5 min each), samples were incubated with SABC‐CY3 (1 µL SABC‐CY3 in 100 µL PBS, 50 µL per section) at 37 °C for 30 min. Finally, sections were washed with 0.5 m PBS (three times, 5 min each), mounted using either a water‐soluble mounting medium or an anti‐fade fluorescence mounting medium, and analyzed using a fluorescence microscope.

### Statistical Analysis

Statistical analysis was performed using GraphPad Prism 8.0 software (GraphPad Software Inc., San Diego, CA, USA). Normality was tested using D'Agostino & Pearson test. When all groups were normally distributed, differences between groups were tested using an unpaired t‐test (for comparison of two groups) or one‐way ANOVA, followed by Holm‐Šídák's Multiple comparisons post‐hoc test (for comparison of more than two groups). When at least one group was not normally distributed, differences between groups were tested using Mann–Whitney test (for comparison of two groups) or Kruskal–Wallis, followed by a Dunn's Multiple comparisons post‐hoc test (for comparison of more than two groups). Correlations were tested using the Spearman's rank correlation coefficient. A p‐value of ≤ 0.05 was considered statistically significant. The following code was used to indicate the level of significance: ^*^ *P* ≤ 0.05; ^**^ *P* ≤ 0.01; ^***^ *P* ≤ 0.001; ^****^ *P* ≤ 0.0001.

## Conflict of interest

The authors declare no conflict of interest.

## Author contributions

M.Y. and Q.W. contributed equally to this work. M.Y., Q.W., D.K., Y.J., and S.W. performed experiments, M.Y. designed and conducted the experiments, analyzed and interpreted the results; M.Y., C.M., R.L., J.G., and J.W. and X.W. supervised the experiments, analyzed the results. M.Y., Q.W., and J.G. wrote the original manuscript. J.G. and X.W. edited and finalized the manuscript. All authors reviewed and approved the final version of the manuscript.

## Supporting information



Supporting Information

Supporting Information

Supporting Information

Supporting Information

## Data Availability

The data that support the findings of this study are available from the corresponding author upon reasonable request.
