# Supplementary material for: Cryptic Splicing of GAP43 mRNA is a Novel Hallmark of TDP‐43‐Associated ALS and AD
Source: Adv Sci (Weinh). 2025 Jun 29;12(36):e12054. doi: 10.1002/advs.202412054 (PMC12463067; doi:10.1002/advs.202412054)
Supplement: Supplementary file 1 — Supporting Information [file ADVS-12-e12054-s004.docx]

**Supplementary Material**

**Cryptic Splicing of *GAP43* mRNA is a Novel Hallmark of** **TDP-43-associated ALS and AD**

Mingming Yang^1,2^†, Qi Wang^1^†, Dongkun Kang^1^, Shijia Wang^3^, Yanli Jiang^1^, Jian-Zhi Wang^1,4^, Chen Ming^3^, Rong Liu^1^, Jianlan Gu^2*^, Xiaochuan Wang^1,4*^

^1^Department of Pathophysiology, School of Basic Medicine, Key Laboratory of Education Ministry/Hubei Province of China for Neurological Disorders, Tongji Medical College, Huazhong University of Science and Technology, Wuhan, Hubei 430030, China

^2^Department of Biochemistry and Molecular Biology, School of Medicine, Key Laboratory of Neuroregeneration and Ministry of Education of Jiangsu, Co-innovation Center of Neuroregeneration, Nantong University, Nantong, Jiangsu 226001, China

^3^Department of Public Health and Medicinal Administration, Faculty of Health Sciences, University of Macau, Macao SAR 999078, China

^4^Hubei Key Laboratory of Cognitive and Affective Disorders, Institute of Biomedical Sciences, School of Medicine, Jianghan University, Wuhan, Hubei 430056, China

†The authors contributed equally to this work

*Corresponding authors, Jianlan Gu, E-mail: ntgjlan@ntu.edu.cn, Xiaochuan Wang, E-mail: wangxiaochuan@hust.edu.cn;

**Supplementary Figures**

**
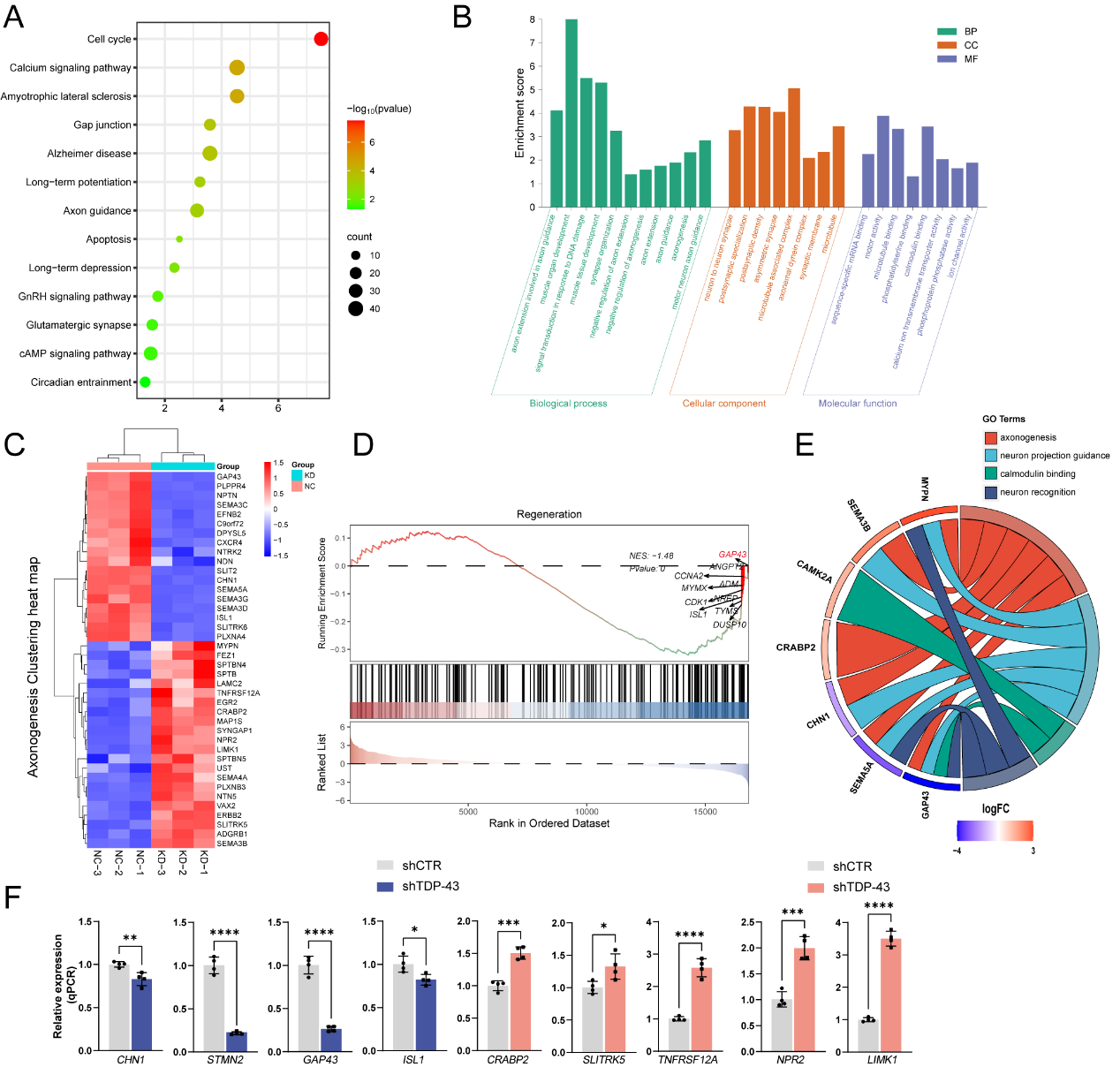
**

**Figure S1. TDP-43 knockdown leads to neuronal synaptic deficits.** (**A**) Dot plot of KEGG analysis of TDP-43 knockdown M17 cells. The analysis was based on the protein counts of the lists ranked by log2FC in the X-axis. (**B**) GO analysis of differentially expressed genes between shCTR and shTDP43. BP, biological processes; CC, cellular component; MF, molecular function. (**C**) Heatmap summary of the top 40 genes changed obviously in axongenesis. *GAP43* was the most significantly changed among the down-regulated genes. (**D**) GSEA analysis of geneset for regeneration. NES, normalized enrichment score; FDR, false discovery rate. Positive and negative NES indicate higher and lower expression in iwt, respectively. (**E**) Chord Diagram of the intersection between genes and the selected pathways, with 4 colors representing axonogenesis (red), neuron projection guidance (light blue), calmodulin binding (green) and neuron recognition (dark blue). The arcs indicate overlapping association between genes and the pathways. (**F**) qPCR analysis of top 9 synapse-related genes changed in transcriptome sequencing of shTDP-43 M17 cells and quantitated (n = 4). Data are mean ± s.e.m.; **P*<0.05; ***P*<0.01; ****P*<0.001; *****P*<0.0001 for shTDP-43 versus shCTR (Unpaired two-tailed Student’s *t* test.).


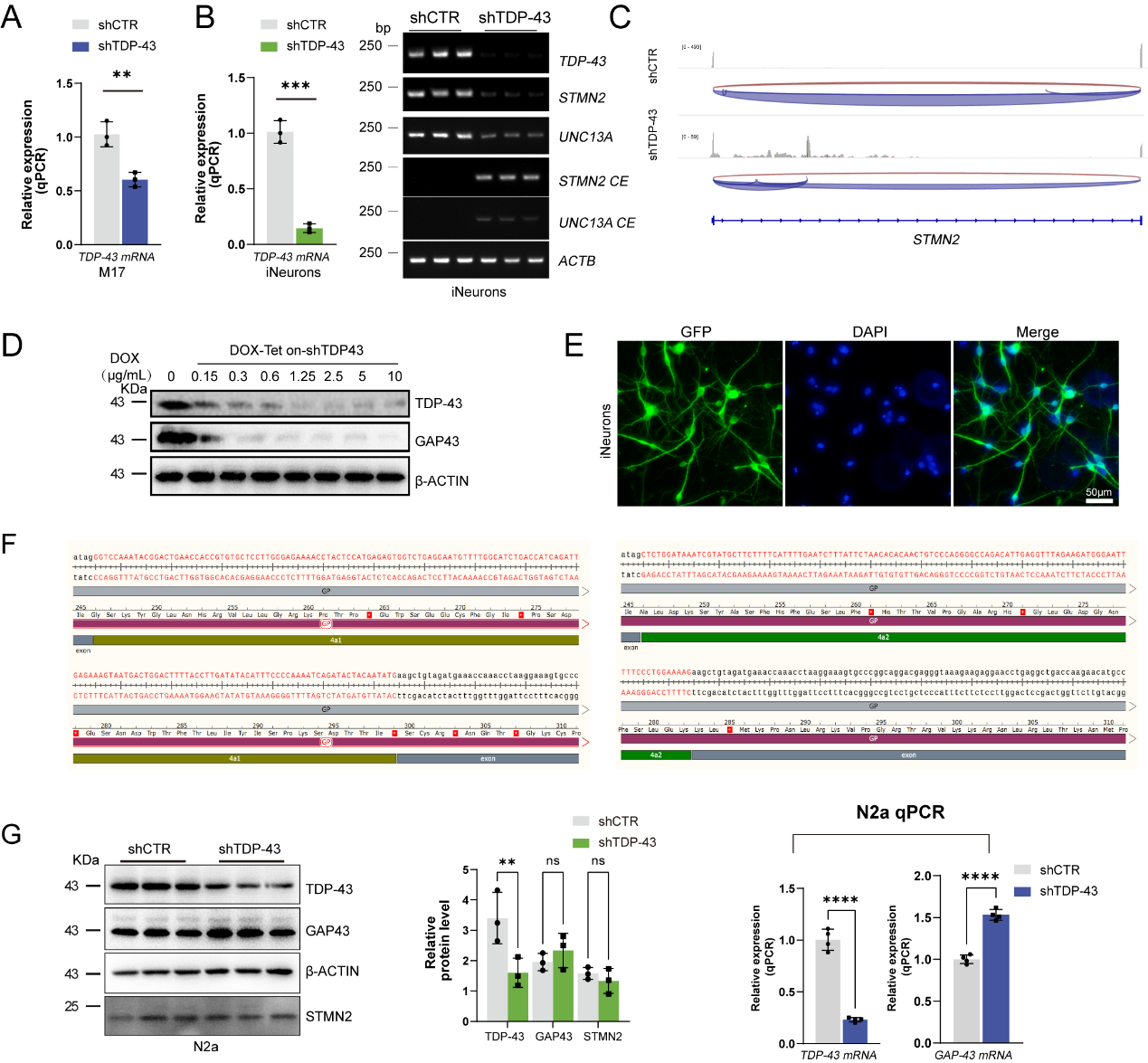


**Figure S2. TDP-43 knockdown in N2a cells does not alter the expression of STMN2 and GAP43.** (**A**) qPCR validates of *TDP-43* transcripts in shTDP-43 M17 cells (n = 3). ***P*<0.01 for shTDP-43 versus shCTR (Unpaired two-tailed Student’s *t* test). (**B**) qPCR validates TDP-43 knockdown (left) and RT-PCR detects *STMN2* and *UNC13A* CE in shTDP-43 iNeurons (n = 3). ****P*<0.001 for shTDP-43 versus shCTR (Unpaired two-tailed Student’s *t* test). (**C**) Visualization of RNA-seq reads mapping to S*TMN2* from shTDP-43 M17 cells. (**D**) Western blotting analysis of TDP-43 and GAP43 in various dosage of DOX-induced shTDP-43 M17 cells. (**E**) Representative images of GFP staining showing the formation of synapses in iNeurons 4 weeks later. (**F**) Graphical representation of the conventional sequences of *GAP43*. *GAP43* contains *4a1* or *4a2* with several stop codons. (**G**) Western blotting and qPCR analysis of GAP43 and STMN2 in shTDP-43 N2a cells and quantitated (n = 3)**.** Data are mean ± s.e.m., ***P*<0.01; *****P*<0.0001 for shTDP-43 versus shCTR; ns, no significance (Unpaired two-tailed Student’s *t* test).

**
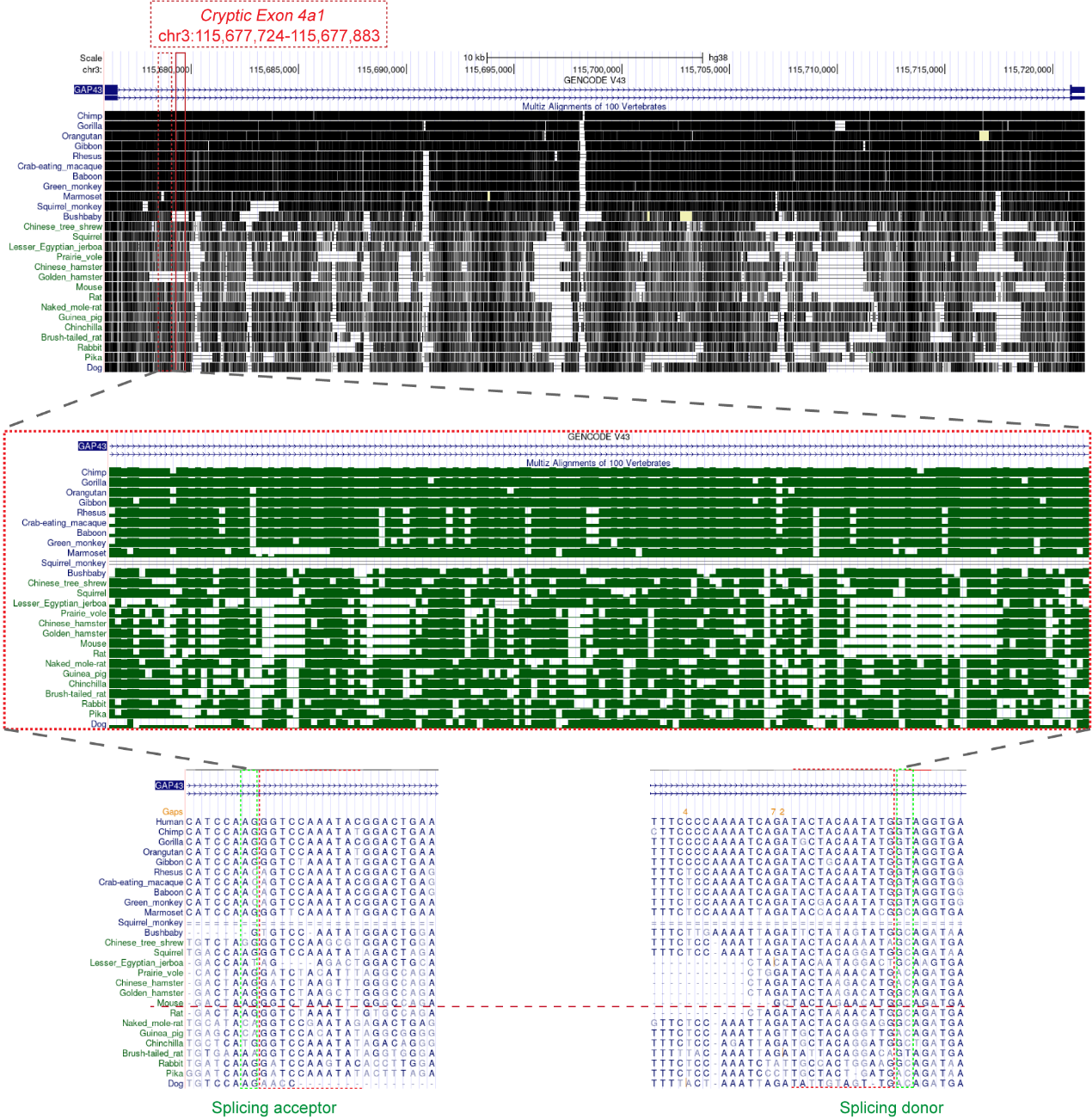
**

**Figure S3. Cryptic exon *4a1* of *GAP43* is conserved in most primates.** Representative graphs show homology alignment of *4a1* sequences of *GAP43* across multiple species. The Multiz Alignments display on UCSC genome browser (http://genome.ucsc.edu). Cryptic exon *4a1* (chr3:115,667,724-115,667,883) of *GAP43* is well conserved among primates. The location of the cryptic exon *4a1* is highlighted in red. The splicing acceptor site upstream of the cryptic exon and splicing donor site downstream of the cryptic exon (highlighted in blue) are conserved only in some primates.


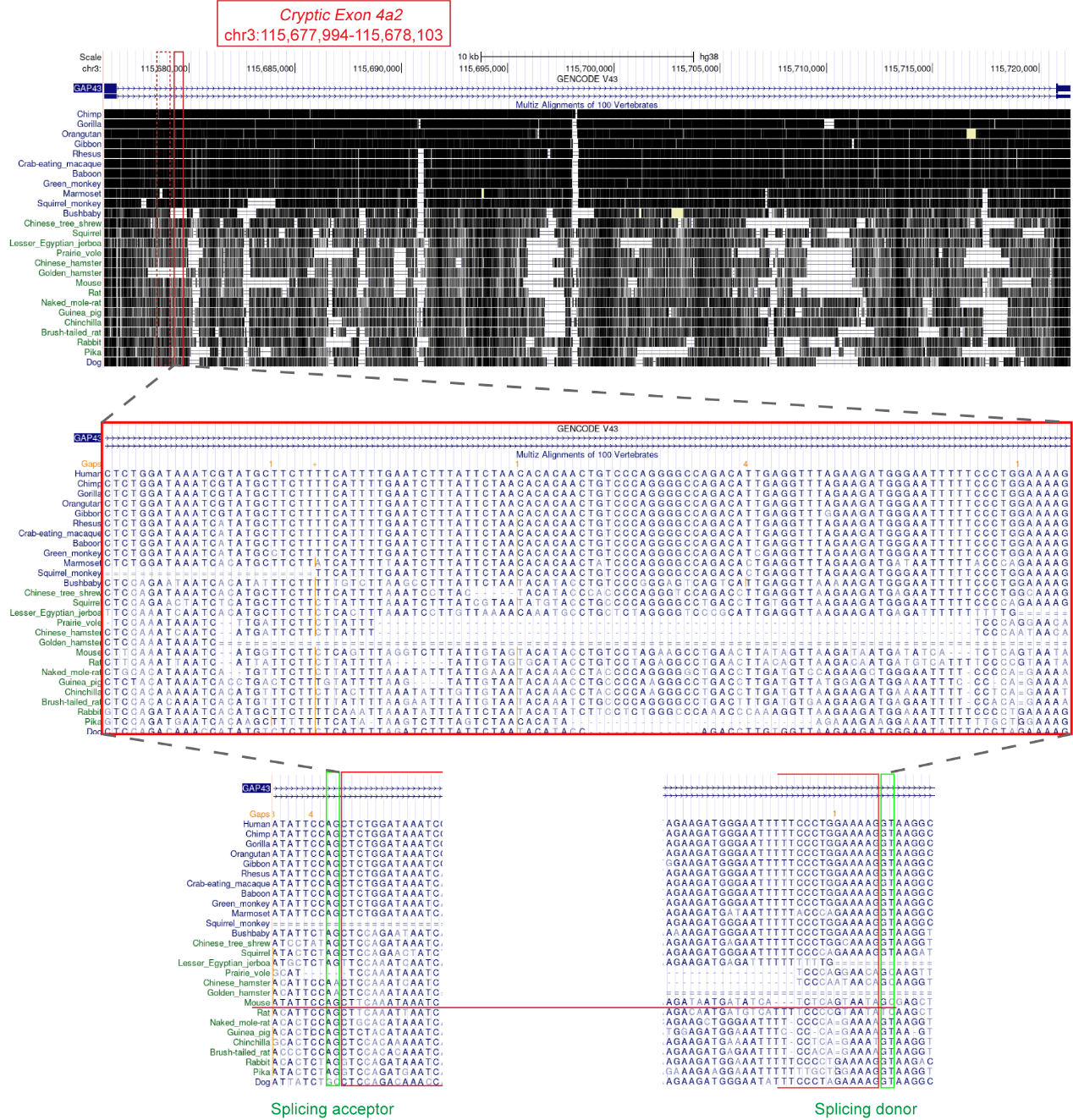


**Figure S4. Cryptic exon *4a2* of *GAP43* is conserved in most primates.** Representative graphs show homology alignment of *4a2* sequences of *GAP43* across multiple species. The Multiz Alignment display on UCSC genome browser (http://genome.ucsc.edu). Cryptic exon *4a2* (chr3:115,677,994-115,678,103) of *GAP43* is well conserved among primates. The location of the cryptic exon *4a2* is highlighted in red. The splicing acceptor site upstream of the cryptic exon and splicing donor site downstream of the cryptic exon highlighted in blue are conserved only in some primates.


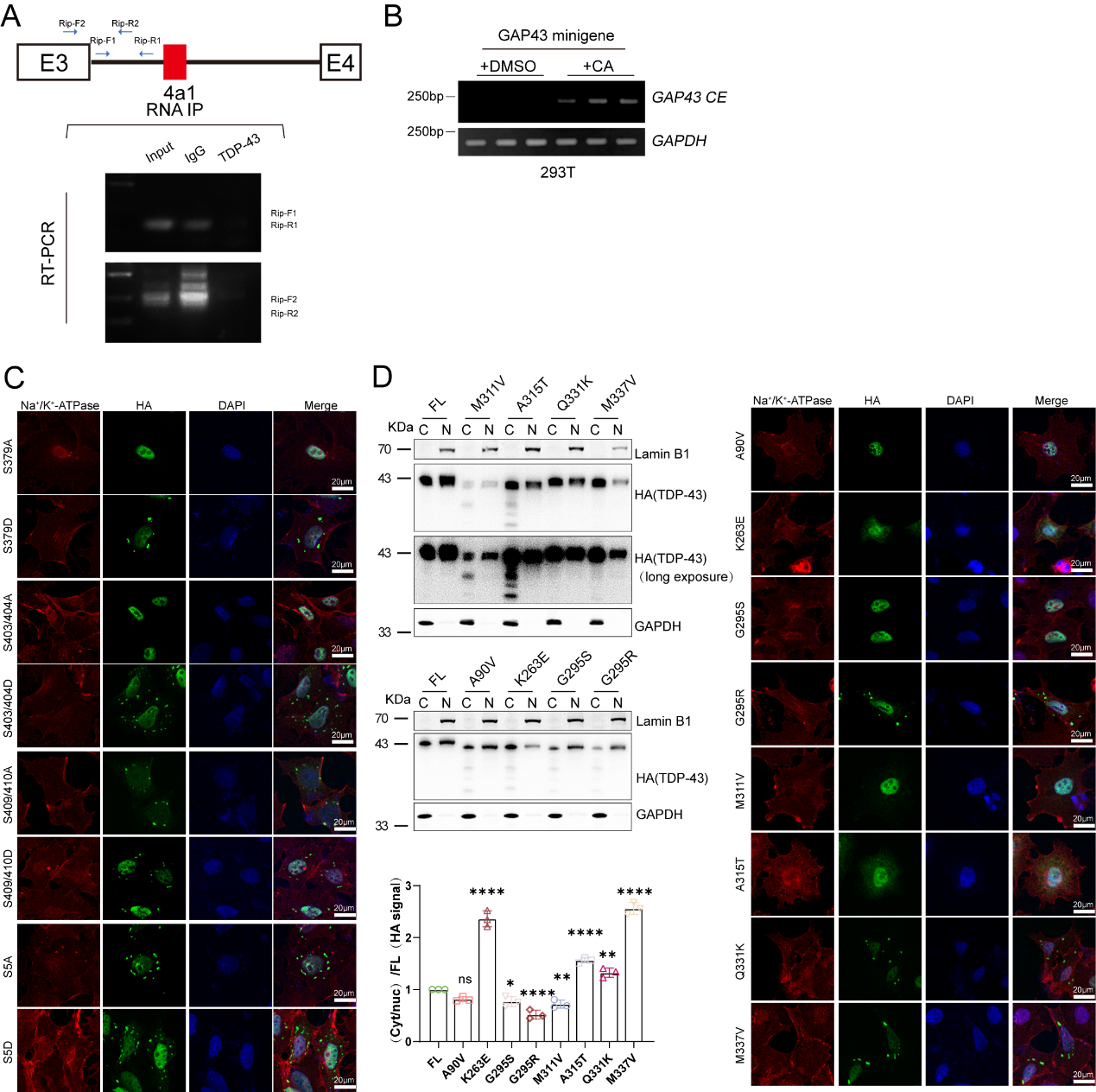


**Figure S5. Phosphorylation and disease-related mutation of TDP-43 promotes its cytoplasmic aggregation.** (**A**) Schematic diagram of the primers for RNA IP (Rip-F1/R1 and Rip-F2/R2). The products of RT-PCR from RNA-IP or cell lysate (Input) were separated by agarose electrophoresis. (**B**) The splicing products of *GAP43* CE in HEK-293T cells transfected with *GAP43* minigene and treated with Calyculin A (CA) were analyzed by RT-PCR. (**C**) Representative images of TDP-43 (anti-HA) in Hela cells transfected with phosphor-mimic or phosphor-blocking mutants of TDP-43. (**D**) Western blotting analysis of TDP-43 (anti-HA) in the cytoplasm (C) or nucleus (N) fractionation in HEK-293T cells transfected with disease-related mutations, and the quantification (n = 3). Data are mean ± s.e.m.; **P*<0.05; ***P*<0.01; *****P*<0.0001 for mutants versus full length (FL) TDP-43; ns, no significance (One-Way ANOVA, Tukey’s multiple comparison). Representative images of TDP-43 (anti-HA) in Hela cells transfected with disease-related mutants of TDP-43 (right panel).

**
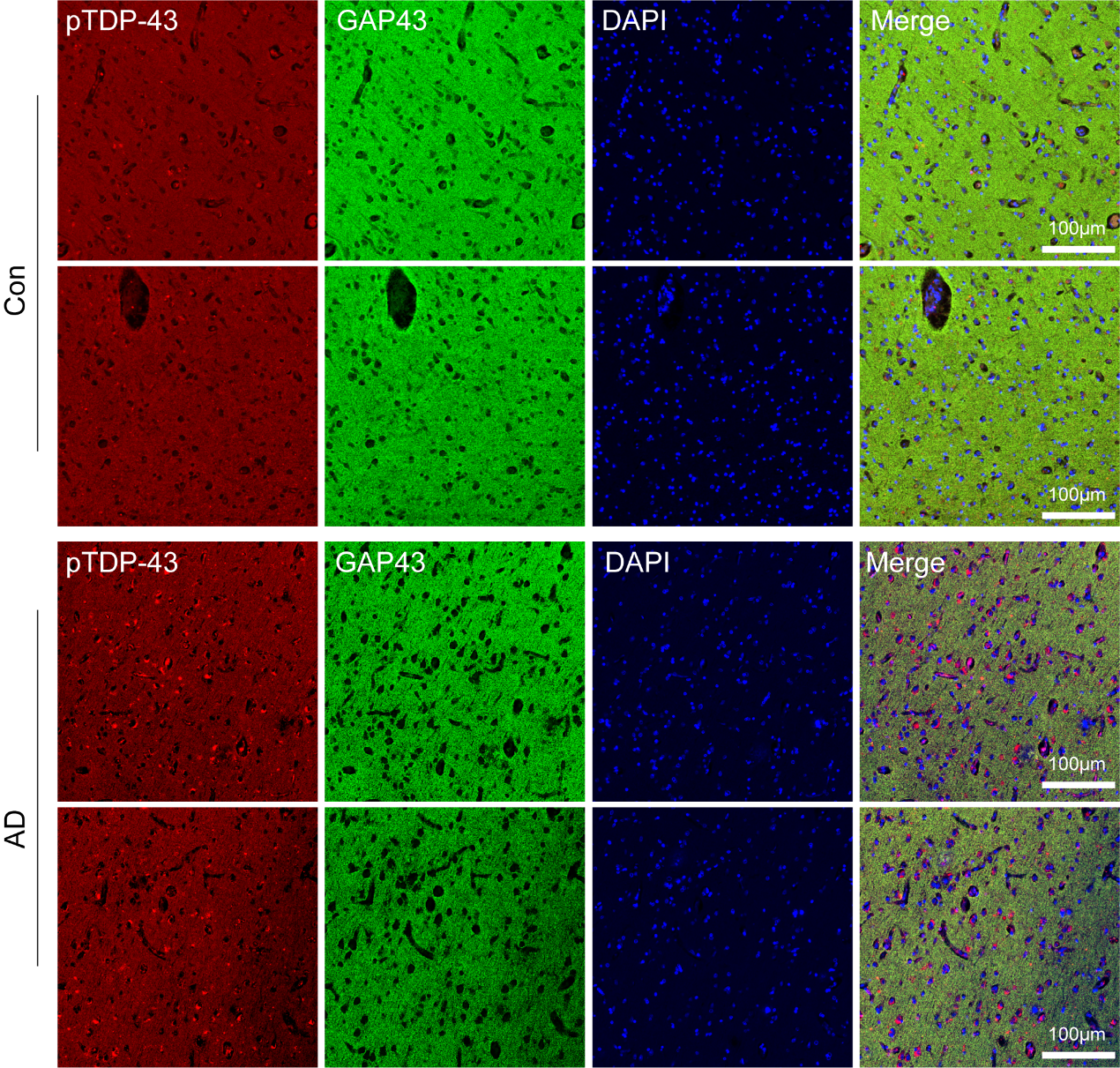
**

**Figure S6. Phosphorylation of TDP-43 is increased in the temporal lobe of AD patients.** Representative images of pTDP-43 (red) and GAP43 (green) staining in temporal sections of normal control and AD patients. Scale bar, 100 µm.

**
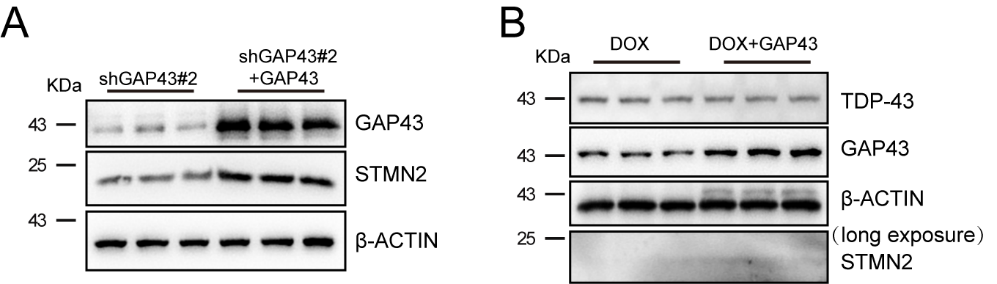
**

**Figure S7. TDP-43-dependent STMN2 decline cannot be rescued by GAP43.** (**A**) Western blotting analysis of GAP43 and STMN2 expression in M17 cells transfected with shGAP43 then ectopic GAP43 expression. (**B**) Western blotting analysis of TDP-43, GAP43 and STMN2 expression in DOX-inducible shTDP-43 M17 cells with ectopic GAP43 expression.

**
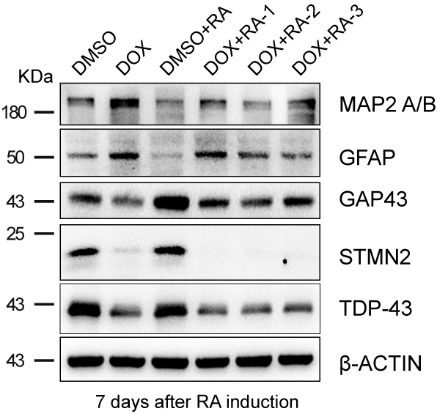
**

**Figure S8. The increased GAP43 expression induced by retinoic acid is inhibited by knockdown of TDP-43.** Western blotting analysis of GAP43 and STMN2 in DOX-inducible shTDP-43 M17 cells transfected with shGAP43, then ectopic GAP43 expression with RA treatment for 7 days.

**
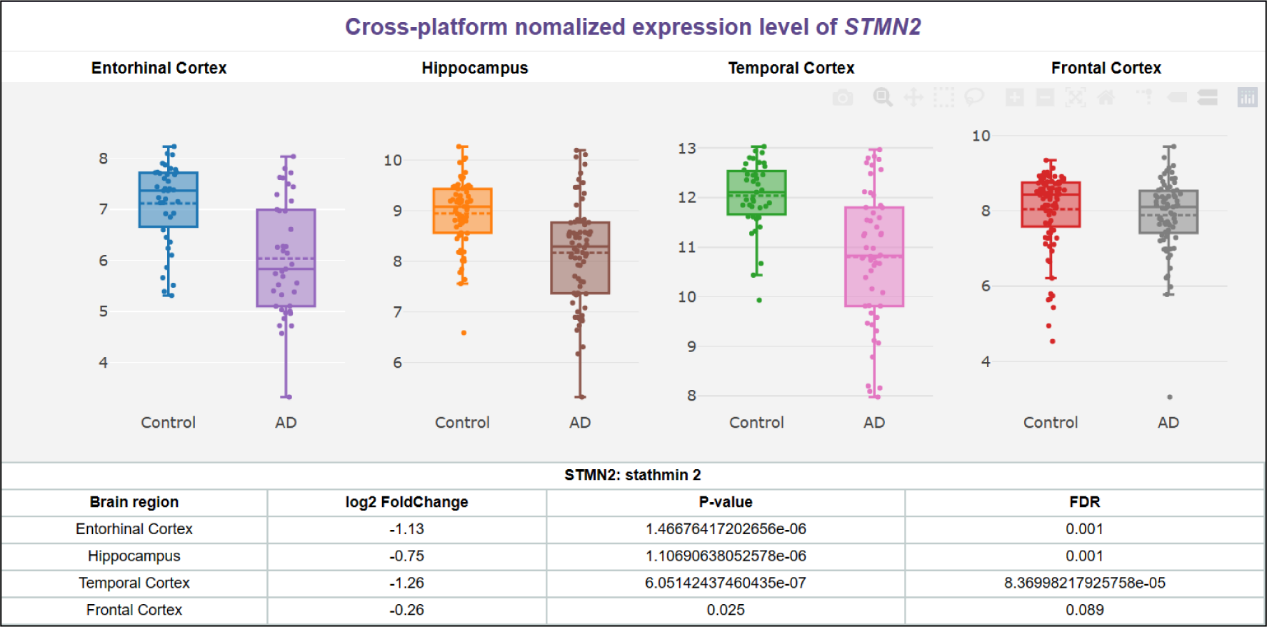
**

**Figure S9. The mRNA of *STMN2* is decreased in various brain regions of AD patients.** Representative images showing the expression of *STMN2* mRNA in different cerebral regions acquired from AlzData dataset.
