# Supplementary material for: Cryptic Splicing of GAP43 mRNA is a Novel Hallmark of TDP‐43‐Associated ALS and AD
Source: Adv Sci (Weinh). 2025 Jun 29;12(36):e12054. doi: 10.1002/advs.202412054 (PMC12463067; doi:10.1002/advs.202412054)
Supplement: Supplementary file 2 — Supporting Information [file ADVS-12-e12054-s001.docx]

**Supplementary Table S1. Human brain tissue of Alzheimer’s disease (AD) and normal control (Con) used in this study**

| **Brain regions** | **Brain Bank Number** | **Case**  **number** | **Group** | **Age at death（y）** | **Sex** | **Braak Stage** | **Brain weight (g)** |
| --- | --- | --- | --- | --- | --- | --- | --- |
| **hippocampus** | 2016CBB007 | Con1 | Con | 83 | M | 0 | 1140 |
|  | 2017CBB013 | Con2 | Con | 74 | M | 0 | 1200 |
|  | 2017CBB025 | Con3 | Con | 70 | F | 0 | 1190 |
|  | 2017CBB034 | Con4 | Con | 98 | F | 0 | 1140 |
|  | 2017CBB021 | Con5 | Con | 72 | M | 0 | 1170 |
|  | 2016CBB029 | AD1 | AD | 83 | M | Ⅳ | 1110 |
|  | 2016CBB021 | AD2 | AD | 99 | F | Ⅴ | 1120 |
|  | 2018CBB002 | AD3 | AD | 73 | M | Ⅲ | 1210 |
|  | 2015CBB31 | AD4 | AD | 79 | M | Ⅵ | 1270 |
|  | 2017CBB046 | AD5 | AD | 71 | F | Ⅳ | 1050 |
| **Brain regions** | **Brain Bank Number** | **Pathology of pTDP-43** | **Group** | **Age at death**  **（y）** | **Sex** | **Braak Stage** | **Brain weight (g)** |
| **Temporal**  **cortex** | 2018CBB009 | negative | Con | 69 | F | 0 | 1280 |
|  | 2019CBB009 | negative | Con | 72 | M | 0 | 1367.2 |
|  | 2017CBB025 | negative | Con | 70 | F | 0 | 1190 |
|  | 2018CBB006 | negative | Con | 84 | F | 0 | 1170 |
|  | 2019CBB009 | negative | Con | 72 | M | 0 | 1367.2 |
|  | 2017CBB046 | positive | AD | 71 | F | Ⅳ | 1050 |
|  | 2018CBB002 | positive | AD | 73 | M | Ⅲ | 1210 |
|  | 2017CBB039 | positive | AD | 71 | F | Ⅳ | 1020 |
|  | 2019CBB021 | positive | AD | 73 | M | Ⅲ | 1234 |
|  | 2015CBB031 | negative | AD | 79 | M | Ⅵ | 1270 |
|  | 2014CBB018 | negative | AD | 88 | M | Ⅵ | none |
