# Supplementary material for: Cryptic Splicing of GAP43 mRNA is a Novel Hallmark of TDP‐43‐Associated ALS and AD
Source: Adv Sci (Weinh). 2025 Jun 29;12(36):e12054. doi: 10.1002/advs.202412054 (PMC12463067; doi:10.1002/advs.202412054)
Supplement: Supplementary file 3 — Supporting Information [file ADVS-12-e12054-s002.docx]

**Supplementary Table S2. Primary antibodies used in this study**

| Antibody | Type | Species | Specificity | Source (Catalog No.) | Usage | Dilution |
| --- | --- | --- | --- | --- | --- | --- |
| Anti-TDP-43 | Poly | R | TDP-43 | ProteinTech (10782–2-AP) | WB | 1:5,000 |
| Anti-Phospho-TDP43 (Ser409/410) | Poly | R | pSer409/410 | ProteinTech (22309-1-AP) | WB | 1:1000 |
| Anti-GAP43 | Mono | R | GAP43_139-238_ | ABclonal (A19055) | WB | 1:3,000 |
| Anti-GAP43 | Poly | R | GAP43 | ProteinTech (16971-1-AP) | WB | 1:3,000 |
| Anti-stathmin2 | Poly | R | STMN2 | ProteinTech (10586-1-AP) | WB | 1:1,000 |
| Anti-stathmin-2 (E1D2M) | Mono | R | STMN2 | CST (38752s) | WB | 1:1000 |
| Anti-N-cadherin | Poly | R | Cadherin | ProteinTech (22018-1-AP) | WB | 1:1000 |
| Anti-CK1ε (H-60) | Poly | R | CK1ε | Santa Cruz (sc-25423) | WB | 1:1000 |
| Anti-CK1δ (E-10) | Mono | M | CK1δ_296-355_ | Santa Cruz (sc-55554) | WB | 1:1000 |
| Anti-Phosphoserine | Poly | R | pSer | Abcam (ab9332) | WB | 1:500 |
| Anti-Lamin B1 | Mono | R | Lamin B1_450-550_ | ABclonal (A11495) | WB | 1:1000 |
| Anti-MAP2 | Poly | R | MAP2 | CST (4542S) | WB | 1:1000 |
| Anti-GFAP (2E1) | Mono | M | GFAP | Santa Cruz (sc-33673) | WB | 1:1000 |
| Anti-HA | Mono | M | HA | ABclonal (AE008) | WB | 1:1000 |
| Anti-β-ACTIN | Mono | R | β-ACTIN | ABclonal (AC026) | WB | 1:10,000 |
| Anti-GAPDH | Mono | R | GAPDH_4-335_ | ABclonal (A19056) | WB | 1:10,000 |
| Anti-GAP43 | Mono | R | GAP43_139-238_ | ABclonal (A19055) | IF/IHC | 1:100 |
| Anti-Phospho-TDP-43 (Ser409/410) | Mono | M | pSer409/410 | ProteinTech (66318-1-Ig) | IF | 1:100 |
| Anti-MAP2 (E-12) | Mono | M | MAP2_1-300_ | Santa Cruz (sc-74419) | IF | 1:100 |
| Anti-Na^+^/K^+^-ATPase | Mono | R | Na^+^/K^+^-ATPase_1-100_ | ABclonal (A11683) | IF | 1:100 |
| Anti-HA | Mono | M | HA | ABclonal (AE008) | IF | 1:100 |
