# Supplementary material for: Cryptic Splicing of GAP43 mRNA is a Novel Hallmark of TDP‐43‐Associated ALS and AD
Source: Adv Sci (Weinh). 2025 Jun 29;12(36):e12054. doi: 10.1002/advs.202412054 (PMC12463067; doi:10.1002/advs.202412054)
Supplement: Supplementary file 4 — Supporting Information [file ADVS-12-e12054-s003.docx]

**Supplementary Table S3. Primers used for RT-PCR/qPCR in this study**

| ***GENE*** | **Primers (5’→3’)** | |
| --- | --- | --- |
|  | **Forward** | **Reverse** |
| *ACTB (HUMAN)* | GCCGCCAGCTCACCAT | TCGTCGCCCACATAGGAATC |
| *GAPDH (HUMAN)* | AATGGGCAGCCGTTAGGAAA | GCCCAATACGACCAAATCAGAG |
| *CHN1 (HUMAN)* | TGTCAGAGAAAAGGGTGCAT | TTTCACGAGCGTCGTAAGGT |
| *NPTN (HUMAN)* | GTGAACCTGCAGATCACGGA | TTCATTGGTCCAGCGTCAGG |
| *ISL1 (HUMAN)* | TCCCTATGTGTTGGTTGCGG | CATTTGATCCCGTACAACCTGA |
| *TNFRSF12A (HUMAN)* | GAGAGAGAAGTTCACCACCCC | CACCTTGGAAGGTTCCCCTG |
| *LIMK1 (HUMAN)* | CCTCCAGAGGGCTAAGTGTTG | GTCTGGTAGTAGCAGTGCCC |
| *CRABP2 (HUMAN)* | CGGGCTAGATCCAGAGAACC | TCAGCATCACATTCACCCCC |
| *SYNGAP1 (HUMAN)* | TGCACATGTCCAACCGGAAG | GGAAGCTGCCTCTCTGAGC |
| *NPR2 (HUMAN)* | AGCTGATGCTGGAGAAGGAG | TGGCGACAACATTTCCCTTG |
| *SLITRK5 (HUMAN)* | TGCAGATGGCAACTGAGGTAA | TGCCGTCCTTTTCCTCACAA |
| *GAP43 4a1* | CTCCAACGGAGACTGGGGAG | TGGTTTCATCTACAGCTTCATATTGTAGTATCTGAT |
| *GAP43 4a2* | CTCCAACGGAGACTGGGGAG | TTCATCTACAGCTTCTTTTCCAGGGAA |
| *GAP43*（F1, R1） | CAGGAAGAAGGCAAGGGACGAG | CATAATTCAGAGCAGGACTTTGTCAT |
| *GAP43*（F2, R2） | AAAGTCCTGCTCTGAATTATGCCACC | GCAACAGGGGCTTCATCCTTCTTAT |
| *GAP43*（F3, R3） | CAGCCAAGGAGGAGCCTAAACAAG | AGGACAGGAAGGGACTTCAGAGTG |
| *GAP43*（minigene） | GAACCACCGTGTGCTCCTTGGG | TCCTCTTCTTTACCCTCGTCCTGC |
| *GAP43* pre mRNA  （Rip-F1, Rip-R1） | TAGAAGATGTTTTCCAGAAATCAAGGG | CTTTCTAGTACTGGCTGCTACATAG |
| *GAP43* pre mRNA  （Rip-F2, Rip-R2） | GAGACTGGGGAGAGCAGCCAAG | TTTGGGAATGTAACCTTAGCTCTTAG |
| *GAP43* pre mRNA  （Rip-F3, Rip-R3） | GGTCCAAATACGGACTGAACC | TAGTCACCTACCATATTGTAGTATCTG |
| GFP-4a1 | GACGGCAACTACAAGACCCGC | TGCTTGTCGGCCATGATATAGACGT |
| Probe 4a1 | UGGUUUCAUCUACAGCUUCAUAUUGUAGUAUCUGAU | |
